# Supramolecular *cis*-“Bis(Chelation)” of [M(CN)_6_]^3−^ (M = Cr^III^, Fe^III^, Co^III^) by Phloroglucinol (H_3_PG)

**DOI:** 10.3390/molecules27134111

**Published:** 2022-06-26

**Authors:** Katarzyna Jędrzejowska, Jedrzej Kobylarczyk, Dorota Glosz, Emilia Kuzniak-Glanowska, Dominika Tabor, Monika Srebro-Hooper, Jakub J. Zakrzewski, Katarzyna Dziedzic-Kocurek, Tadeusz M. Muzioł, Robert Podgajny

**Affiliations:** 1 Faculty of Chemistry, Jagiellonian University in Krakow, Gronostajowa 2, 30-387 Kraków, Poland; katarzyna.jedrzejowska@doctoral.uj.edu.pl (K.J.); jedrzej.kobylarczyk@ifj.edu.pl (J.K.); doglosz@wp.pl (D.G.); kuzniak.emilia@gmail.com (E.K.-G.); dominika.tabor@student.uj.edu.pl (D.T.); monika.srebro@uj.edu.pl (M.S.-H.); jakub.j.zakrzewski@doctoral.uj.edu.pl (J.J.Z.); 2 Institute of Nuclear Physics PAN, Radzikowskiego 152, 31-342 Kraków, Poland; 3 Marian Smoluchowski Institute of Physics, Jagiellonian University, Łojasiewicza 11, 30-348 Krakow, Poland; k.dziedzic-kocurek@uj.edu.pl; 4 Faculty of Chemistry, Nicolaus Copernicus University in Toruń, Gagarina 7, 87-100 Toruń, Poland; tmuziol@umk.pl

**Keywords:** hydrogen bond, cyanidometallates, co-crystals, molecular tectons, molecular synthons, Hirshfeld analysis, ETS-NOCV analysis

## Abstract

Studies on molecular co-crystal type materials are important in the design and preparation of easy-to-absorb drugs, non-centrosymmetric, and chiral crystals for optical performance, liquid crystals, or plastic phases. From a fundamental point of view, such studies also provide useful information on various supramolecular synthons and molecular ordering, including metric parameters, molecular matching, energetical hierarchy, and combinatorial potential, appealing to the rational design of functional materials through structure–properties–application schemes. Co-crystal salts involving anionic *d*-metallate coordination complexes are moderately explored (compared to the generality of co-crystals), and in this context, we present a new series of isomorphous co-crystalline salts (PPh_4_)_3_[M(CN)_6_](H_3_PG)_2_·2MeCN (M = Cr, **1**; Fe, **2**; Co **3**; H_3_PG = phloroglucinol, 1,3,5-trihydroxobenzene). In this study, **1**–**3** were characterized experimentally using SC XRD, Hirshfeld analysis, ESI-MS spectrometry, vibrational IR and Raman, ^57^Fe Mössbauer, electronic absorption UV-Vis-NIR, and photoluminescence spectroscopies, and theoretically with density functional theory calculations. The two-dimensional square grid-like hydrogen-bond {[M(CN)_6_]^3−^;(H_3_PG)_2_}_∞_ network features original {[M(CN)_6_]^3−^;(H_3_PG)_4_} supramolecular *cis*-bis(chelate) motifs involving: (i) two double cyclic hydrogen bond synthons M(-CN⋅⋅⋅HO-)_2_Ar, {[M(CN)_6_]^3−^;**H_2_**PGH}, between *cis*-oriented cyanido ligands of [M(CN)_6_]^3−^ and resorcinol-like face of H_3_PG, and (ii) two single hydrogen bonds M-CN⋅⋅⋅HO-Ar, {[M(CN)_6_]^3−^;**H**PGH_2_}, involving the remaining two cyanide ligands. The occurrence of the above tectonic motif is discussed with regard to the relevant data existing in the CCDC database, including the multisite H-bond binding of [M(CN)_6_]^3−^ by organic species, mononuclear coordination complexes, and polynuclear complexes. The physicochemical and computational characterization discloses notable spectral modifications under the regime of an extended hydrogen bond network.

## 1. Introduction

Hexacyanidometallates of *d* block metal ions belong to the most versatile and common building blocks in the construction of functional molecular materials within the frame of various synthetic strategies. Based on their individual properties resulting from the valence electronic structure (magnetic, optical, redox reactivity, communicative molecular orbital system), polynuclear complexes are considered in the construction of switchable materials such as molecular magnets, nanomagnets, and photomagnets or solar energy converting units. These features are very often combined and enhanced with porosity, conductivity, or luminescence under the common flag of the Prussian Blue Analogues (PBAs) family and the related species of various dimensionality [1,2,3].

Nevertheless, going beyond the bimetallic or trimetallic building block approach toward coordination-based networks, another broad field has emerged, focusing on the development of an alternative molecular networks library. As in the case of PBAs, organic and inorganic parties in such networks meet together in shaping their properties and functionality; however, within the current scenery, the [M(CN)_6_]^3−^ anions (being a representant of the broader family of anions) and “non-innocent” organic molecules are combined into hybrid supramolecular architectures through dominant non-covalent interactions. Several interesting groups of hybrid networks have been developed or at least suggested, which can be classified as real salts, double salts, or co-crystal salts, including their solvated analogues [4]. Firstly, a broad family of organic–inorganic perovskites was enriched with [A^I^][B]_2_[M^III^(CN)_6_]·solv (A^+^ = alkali metal cation, B^+^ = alkyl organic cation) species, in which B^+^ are located within the more or less regular cavities formed by the framework of electrostatically bound A^+^ and [M(CN)_6_]^3−^ [5,6,7,8,9]. The significant freedom of reorientation shown by B^+^ cations together with their symmetry and dipolar moment was exploited to imply the occurrence of thermal phase transitions between ordered/disordered states. As a result, notable switchable behavior might be observed for dielectric properties, non-centrosymmetry related properties (e.g., ferro-, pyro-, and piezoelectricity, second harmonic generation and even chirality), symmetry-independent third-harmonic generation (THG), and photoluminescent performance if appropriate species are involved [9]. Alternative molecular antiperovskite architectures were realized via reverse site occupation, hosting [Co(CN)_6_]^3−^ anions at the cavities formed within {(MF_6_)(H_2_dabco)_3_}^3+^ (M = Al^3+^, Cr^3+^, or In^3+^; dabco = 1,4-diazabicyclo[2.2.2]octane) and {(MF_6_)(H_2_pip)_3_}^3+^ (M = Al^3+^ or Cr^3+^; pip = piperazine) frameworks [10]. Secondly, [M(CN)_6_]^3−^ anions were combined with the specially preprogrammed bis(amidinium) organic dications of the dedicated directional distribution of N^+^-H hydrogen bond donors, which resulted in a series of tectonic networks stabilized by charge-assisted hydrogen bonds [11,12], some of them featuring reversible solvent uptake and post-crystallization structural transformations [13,14]. The underlaying crystal phases also gave rise to a series of epitaxially grown core–shell crystalline composites [15], considered molecular waveguides exploiting luminescent properties [16]. Thirdly, planar organic cations, e.g., tetrathiafulvalene (TTF) derivatives, engaged hexacyanidometallates into the switching of charge ordering and conductivity phenomena upon structural-phase transition [17,18]. Finally, selected co-crystalline type phases combining [M(CN)_6_]^3−^ anions and planar neutral π-acidic species, e.g., naphtalenediimide (NDI) derivatives [19] and 1,4,5,8,9,12-hexaazatriphenylenehexacarbonitrile (HAT(CN)_6_), showed charge transfer properties [20], whereas specially preprogrammed pyrimidine derivatives [21,22] were studied from the standpoint of surface-enhanced anion binding.

The current study is dedicated to new *d*-metallate-based supramolecular synthons that might be of importance in the design of functional molecular platforms in solution and in the solid-state. As a counterpart, we selected phloroglucinol (H_3_PG; 1,3,5-trihydroxobenzene), a triangular hydrogen bond donor widely tested in the formation of hydrogen-bonded architectures [23,24,25,26,27,28,29,30,31,32,33,34,35,36,37,38,39]. Its resulting functional character is well documented in the context of topological reactivity [24], combinatorial or modular synthetic strategies [25,35], molecular recognition and selective binding [31,34,39], proton disorder [26], drugs fabrication improvement [27], luminescence switching [28], switchable magnetic properties [23], chiral properties and photonic materials [36], and general molecular organization [23,29,30,31,32,33,34,37,38]. In particular, molecular recognition and selective binding of H_3_PG and other associated resorcinol and naphtalenediol-type co-formers involved various polynuclear d-metal ions clusters serving as the molecular hosts [31,34,39,40,41,42,43,44,45,46]. However, anionic d-metallates or f-metallates were moderately represented in these studies, counting some examples of pyridine–dicarboxylate complexes forming the hydrogen-bonded networks with pyridinium and polypyridinium cations [34,43,45,46]. 

In this paper, we present a new series of hybrid organic–inorganic isomorphous co-crystal salts (PPh_4_)_3_[M(CN)_6_](H_3_PG)_2_·2MeCN (M = Cr, **1**; Fe, **2**; Co **3**). As a novel contribution to the field of multicomponent molecular architectures, our structural, physicochemical, and computational studies describe the original {[M(CN)_6_]^3−^;(H_3_PG)_4_} supramolecular *cis*-bis(chelate) motifs involving typical single M-CN⋅⋅⋅HO-Ar and double cyclic M(-CN⋅⋅⋅HO-)_2_Ar hydrogen-bond synthons.

## 2. Results and Discussion

### 2.1. Structural Studies

**Figure 1:**
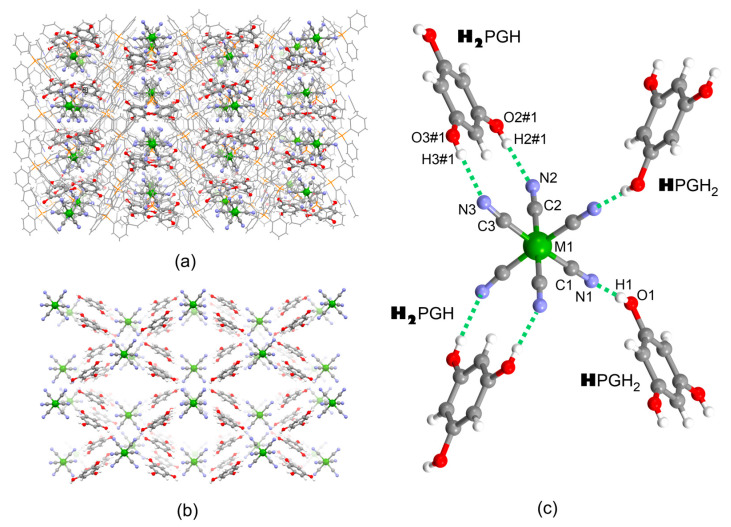
Crystal structure of **1–3:** (**a**) hydrogen-bonded {[M(CN)_6_]^3−^;{(H_3_PG)_2_} layers spread along the *ab* crystallographic plane; (**b**) perpendicular view of hydrogen-bonded {[M(CN)_6_]^3−^;(H_3_PG)_2_} layers along *c* crystallographic direction (PPh_4_^+^ cations and MeCN solvent molecules omitted for clarity); (**c**) supramolecular hydrogen-bonded ***cis*-“bis(chelate)”** {[M(CN)_6_]^3−^(**H_2_**PGH)_2_(**H**PGH_2_)_2_} as a part of the hydrogen-bonded single layer (for metric parameters see Table 1). Legend: green—Cr, Fe or Co, grey—C, blue—N, red—O, orange—P, white—H; protons involved in hydrogen bonds with one [M(CN)_6_]^3−^ are indicated in bold.

Compounds **1**–**3** crystallized in the monoclinic system, space group C2/c (Table S1). The uniformity of the powder samples and the identity of the crystals examined with SC XRD were confirmed by PXRD (Figure S1, Supplementary Materials). The crystal structures consist of PPh_4_^+^ cations, hexacyanidometallate [M(CN)_6_]^3−^ anions, phloroglucinol (H_3_PG) co-former molecules, and solvent molecules MeCN. Asymmetric units contain 1 and ½ PPh_4_^+^, 1 H_3_PG, ½ [M(CN)_6_]^3−^ anion, and 1 MeCN molecule (Figure S2), and the most important bond lengths and angles are shown in Table S2. [M(CN)_6_]^3−^ and H_3_PG form an exclusive hydrogen-bonded 2D subnetwork {[M(CN)_6_]^3−^;(H_3_PG)_2_}_∞_, which coexists with another subnetwork formed by organic cations and solvent molecules (Figure 1a,b). The {[M(CN)_6_]^3−^;(H_3_PG)_2_}_∞_ subnetwork reveals a rhombus square grid-like topology, where [M(CN)_6_]^3−^ hydrogen-bond acceptors are located in its nodes, and H_3_PG hydrogen bond donors act as linear linkers. Each [M(CN)_6_]^3−^ anion forms four hydrogen bond contacts with four neighboring H_3_PG molecules (Figure 1c): two double cyclic ring-type pattern $R_{2}^{2}\left(12\right)$ synthons with two H_3_PG, {[M(CN)_6_]^3−^;**H_2_**PGH} and two single synthons of the linear pattern *D* with the remaining two H_3_PG molecules, {[M(CN)_6_]^3−^;**H**PGH_2_} [47]. Importantly, the original double synthon is formed owing to structural and electronic matching between the *cis*-dicyanido fragment of the complex and resorcinol-like [48] (1,3-dihydroxobenzene) fragment of H_3_PG, as a vivid example of the realization of the *molecular tectonics* concept [12]. The interatomic distances and the related angles within both synthons are collected in Table 1. The observed N⋅⋅⋅O and N⋅⋅⋅H separations, together with the related almost linear N⋅⋅⋅H-O angles, allow us to classify them as moderate-to-strong hydrogen-bonding interactions [49] (see also calculated interaction energies presented in the Section 2.4 below). Among the N⋅⋅⋅O and N⋅⋅⋅H distances, the shortest ones are noted in the single {[M(CN)_6_]^3−^;(**H**PGH_2_)} synthon along with the whole **1**–**3** series, whereas those in the {[M(CN)_6_]^3−^;(**H_2_**PGH)} synthon are slightly longer and notably diversified, which is most probably due to steric effects that might accompany the formation of such a complex motif. The double synthons are almost planar, with a ca. 10° angle between the H_3_PG plane and the plane formed by the *cis*-dicyanido fragment of [M(CN)_6_]^3−^. The described spatial orientation of all four synthons around the central [M(CN)_6_]^3−^ within the {[M(CN)_6_]^3−^;(**H_2_**PGH)_2_(**H**PGH_2_)_2_} structural fragment shown in Figure 1c is identical to the distribution of ligands in canonical *cis* stereoisomers of bis(chelated) six-coordinate *d* metal ion complexes [ML_2_A_2_] (L = bidentate chelating ligands, A = monodentate ligands), and thus we suggest to describe such aggregation as *the non-covalent cis-bischelation*. Moreover, the formation of the double synthons leads to the notable deformation of the [M(CN)_6_]^3−^ complex from octahedron following the spatial demands imposed by the distribution of the -OH groups in H_3_PG. The C2-M1-C3 and N2⋅⋅⋅M1⋅⋅⋅N3 angles in all compounds are close to 85° and 82°, respectively, notably smaller compared to other close-to-right angles; this feature resembles the standard biting angles of ca. 72–75° observed in the complexes with the flat chelating ligands (e.g., *o*-phenanthroline). Furthermore, the M1-C3-N3 angles involved in the double synthons are visibly deviated from linearity, unlike the M1-C1-N1 and M1-C2-N2 ones. The M-C and C-N bond lengths and M-C-N angles in [M(CN)_6_]^3−^, as well as all metric parameters of H_3_PG, are in good agreement with the statistical values observed in the CCDC database. The overall deformation of [M(CN)_6_]^3−^ complexes might be represented by shape measures *S*_OC-6_ (Table S3) using the CShM method [50].

The molecular environment of the tectons engaged in the hydrogen-bond networks is completed by the oligomeric arrays of PPh_4_^+^ cations accompanied by some MeCN molecules; these components form multiple weak C-H⋅⋅⋅A interactions (A = O atoms and ring system of H_3_PG, N atoms of [M(CN)_6_]^3−^) in the regions not involved in the typical hydrogen bonds (Figure S3). The {PPh_4_^+^}_∞_ 3D subnetwork itself provides substantial structural stabilization through so-called multiple phenyl embrace (MPE) motifs, here realized mainly by the sextuple phenyl embrace (SPE) or offset sextuple phenyl embrace (OSPE) and other hybrid patterns, with the shortest P⋅⋅⋅P distances of 6.2, 6.6, and 7.3 Å in all structures [51,52] (Figure S4). 

The non-covalent *cis*-bis(chelated) {[M(CN)_6_]^3−^(**H_2_**PGH)_2_(**H**PGH_2_)_2_} motif observed in the crystal structures of **1**–**3** obeys the local *C*_2_ symmetry axis passing through the M1 center (this local axis converges with the lattice *C*_2_ axes). Again, as in the case of the *cis*-bis(chelated) [M(L_2_A_2_)] building blocks, this feature might be important from the standpoint of the molecular design of chiral and non-centrosymmetric molecular materials. Such local arrangement is a rare feature among architectures hosting non-bridged [M(CN)_6_]^3−^ anions and might be realized with the tris(2-aminoethyl)amine or tris(2-pyridylmethyl)amine ligand of the neighboring cationic complex [53]. On the contrary, more examples of *trans*-bischelation were found in the structural database, including synthons with multiple hydrogen bond donors as large cyclic alkane-polyammonium organic cations [54] or alkane-polyamine ligands of other metal complexes in the structure [55,56,57]. Interestingly, the hydrogen-bonded trischelation involving bis(amidinium) dications and [M(CN)_6_]^3−^ tectons might be distinguished along with the series of honeycomb 2D tectonic networks reported by Ferlay and Hosseini [12,13,14], important from the viewpoint of porosity design. However, these synthons were not considered in terms of local symmetry by these authors. Our 2D square-grid hydrogen-bonded arrangement provides a new topological solution, an alternative to the 2D honeycomb architecture based on H_3_PG and [Co_2_Fe_2_(CN)_6_(tp*)_2_(bpy*)_4_]^2+^ cationic block (tp* = hydrotris(3,5-dimethylpyrazol-1-yl)borate, bpy* = 4,4′-dimethyl-2,2′-bipyridine), and involved in the thermal and light-induced electron-transfer-coupled spin transitions (ETCST) [23]. 

It must be underlined that the described hydrogen-bonded network definitely dominates the overall architecture in **1**–**3**, which results in the significant modification of the [M(CN)_6_]^3−^ surroundings compared to that in the crystal structures of the (PPh_4_)_3_[Cr(CN)_6_]·2H_2_O (refcode SEGFAM) [58] and (PPh_4_)_3_[Fe(CN)_6_]·7H_2_O (refcode VOLVEZ) [59]. This modification consists of the saturation of hydrogen-bonding connections around [M(CN)_6_]^3−^ exploiting much stronger Brønsted ring-OH acid compared to H-OH or ring-C-H groups (Figure S5), with a high potential to change the spectroscopic characteristics of **1**–**3** relative to their parent compounds (see below).

### 2.2. Hirshfeld Analysis

**Figure 2:**
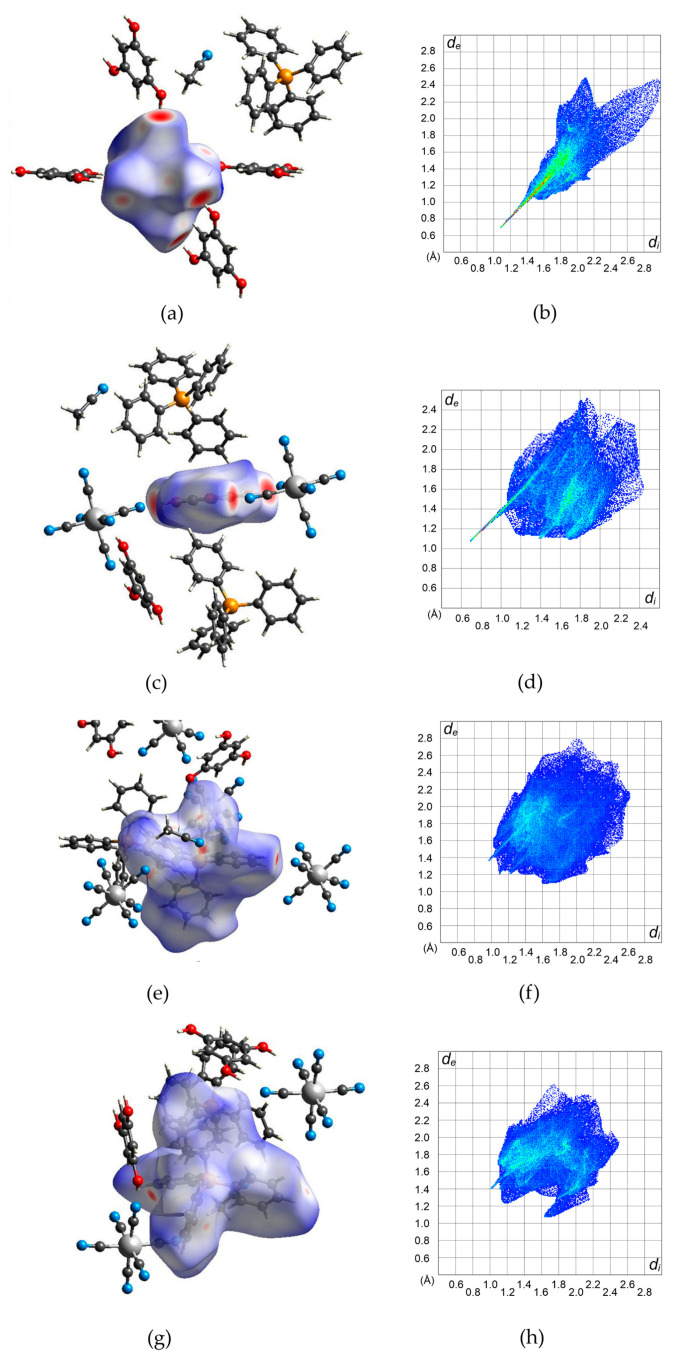
Hirshfeld surfaces (**left column**) of the molecular building blocks in **2** and corresponding fingerprints (**right column**) for all interactions: (**a**,**b**)–[Fe(CN)_6_]^3−^; (**c**,**d**)–H_3_PG; (**e**,**f**)–P(1)Ph_4_^+^; (**g**,**h**)–P(2)Ph_4_^+^. The detailed images for all individual interactions are given in the Supplementary Materials (Figures S6, S7, S9 and S10).

**Figure 3:**
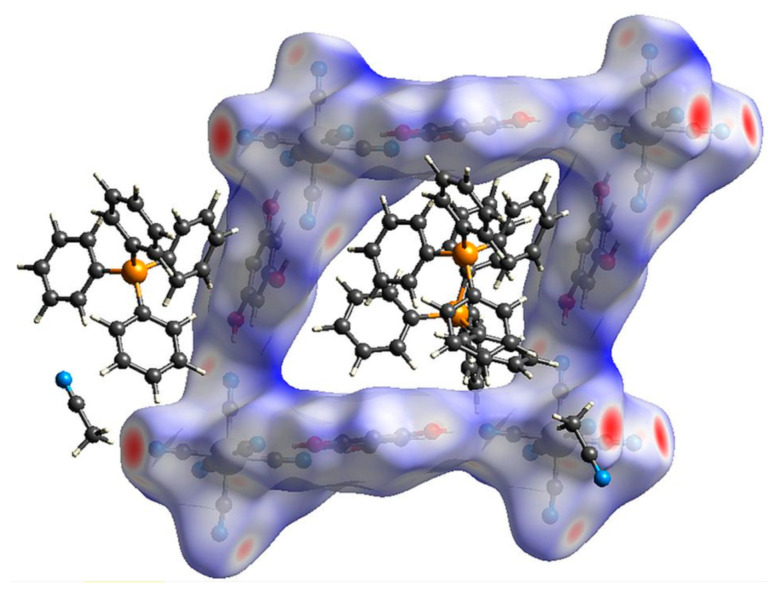
Hirshfeld surface presenting contacts formed by the anion subnetwork grid.

The square-grid-like anionic subnetwork is possible due to two synthons based on moderate-to-strong hydrogen bonds. These features can be observed on the Hirshfeld surfaces [60,61,62] generated for the [Fe(CN)_6_]^3−^ anion and H_3_PG molecule (Figure 2a–d). They emerge as red spots marking distances shorter than a sum of van der Waals radii and also as spikes on corresponding fingerprints. This analysis also shows that in the case of the [Fe(CN)_6_]^3−^ unit, N⋅⋅⋅H interactions prevail (74.4%) (Figure S6). For H_3_PG, it is only 14.7% of created contacts, but they are the shortest ones and correspond to hydrogen bonds, whereas O⋅⋅⋅H distances are more numerous (23.1%) but much longer, and the tiny spike at (1.4, 1.1) corresponds to two C-H⋅⋅⋅O hydrogen bonds (Figure S7). Both synthons can be clearly visible in Figure S8. The interface is formed by H⋅⋅⋅N and H⋅⋅⋅C atoms for double synthons and H⋅⋅⋅N for single ones. For H_3_PG, the most numerous (38.2%) H⋅⋅⋅H interactions are created with surrounding PPh_4_^+^ cations. The C-H⋅⋅⋅N hydrogen-bonds trap PPh_4_^+^ units in the anionic subnetwork, which supports the compartmentalization of the cations despite electrostatic repulsion. Dance et al. explained this effect by aromatic rings embraces [51] (see above), which can also be nicely identified on Hirshfeld surfaces (Figure 2e–h). These contacts are formed by hydrogen and carbon atoms of two interacting PPh_4_^+^ units (Figures S9 and S10). Due to asymmetric unit content, the column pattern is given as ⋅⋅⋅P1-P1-P2⋅⋅⋅ and there are two interfaces between two P1 moieties and P1 and P2 units. Figure S11 present all interfaces involved in these interactions with prevailing H⋅⋅⋅C and H⋅⋅⋅H contacts. Mutual orientation of phenyl rings involved in this embrace points to SPE or OSPE motifs. There are also some red spots on the Hirshfeld surface which were identified as C-H⋅⋅⋅N hydrogen bonds with [Fe(CN)_6_]^3−^ anions and acetonitrile molecules (Figures S9g and S10g). They occur as small spikes at ca. (0.95, 1.35) and ca. (1, 1.4) (Figures S9h and S10h). Hence, these interactions are involved in the network stabilization and interactions between both sublattices (Figure 3). The positions of (PPh_4_)^+^ cations and the surface color indicate that weak interactions prevail, corresponding to X⋅⋅⋅H (X = H, C, N) created by the [Fe(CN)_6_]^3−^ anion and H_3_PG involved in π-π interactions between strongly inclined rings.

### 2.3. ESI-MS

**Figure 4:**
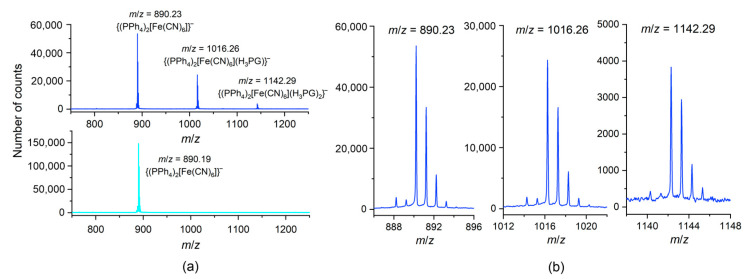
The ESI–MS spectra in the negative ionization mode for **2** (blue) and for (PPh_4_)_3_[Fe(CN)_6_]·6H_2_O (cyan) as a reference: (**a**) the *m*/*z* range of 750^–^–1250^–^ showing the peak-sets assigned to {(PPh_4_)_2_[M(CN)_6_]}^–^, {(PPh_4_)_2_[M(CN)_6_](H_3_PG)}^–^, and {(PPh_4_)_2_[Fe(CN)_6_](H_3_PG)_2_}^–^ aggregates; (**b**) the details of the relevant isotopic patterns. The spectra are fully representative for the whole series **1**–**3**, based on the perfect fit of the individual isotopic patterns.

The ESI-MS spectrograms in the negative ionization mode measured for MeOH solutions of **1**–**3** do not differ significantly from each other. All of them consistently exhibit the presence of three mono-negative peak-sets assignable to the salt-like {(PPh_4_)_2_[M(CN)_6_]}^–^ aggregates (*m/z* = 886.24^–^ for **1**, 890.23^–^ for **2**, and 893.23^–^ for **3**) (as a dominating feature), and to two progressive co-crystal-like {(PPh_4_)_2_[M(CN)_6_](H_3_PG)} ^–^ (*m/z* = 1012.27^–^ for **1**, 1016.26^–^ for **2**, and 1019.27^–^ for **3**) and {(PPh_4_)_2_[Fe(CN)_6_](H_3_PG)_2_}^–^ (*m/z* = 1138.32^–^ for **1**, 1142.29^–^ for **2**, and 1145.30^–^ for **3**) aggregates (Figure 4 and Figure S12). A similar progression in positive ionization mode involves the mono-positive {(PPh_4_)_4_[M(CN)_6_]}^+^ (*m/z* = 1565.48^+^ for **1**, 1569.47^+^ for **2**, and 1572.45^+^ for **3**) (as a dominating feature), {(PPh_4_)_4_[M(CN)_6_](H_3_PG)}^+^ (*m/z* = 1691.49^+^ for **1**, 1695.52^+^ for **2**, and 1698.48+ for **3**), and {(PPh_4_)_4_[Fe(CN)_6_](H_3_PG)_2_}^+^ (*m/z* = 1821.50^+^ observed only for **2**) aggregates (Figure S13). The absence of {(PPh_4_)_4_[Cr(CN)_6_](H_3_PG)_2_}^+^ and {(PPh_4_)_4_[Co(CN)_6_](H_3_PG)_2_}^+^ motifs might be attributed to competitive fragmentation events. Interestingly, in the spectral *m*/*z* range of 1980^+^–2850^+^, the Fe congener **2** shows numerous peak-sets of the isotopic patterns assignable to the aggregates of the general formula {(PPh_4_)*_x_*[Fe(CN)_6_]*_y_*(H_3_PG)*_z_*}*^n^*^+^, (*x*, *y*, *z*–small natural numbers; *n* = 2, 3). Among them, we recognized the distinct peak-sets located at every 63 *m*/*z* unit and showed the component lines separation of *m*/*z* = 0.5 units, attributable to Fe-containing aggregates of double positive charge. The other peak-sets were located at every 42 *m*/*z* unit and exhibited the component lines separation of *m*/*z* = 0.33, and were tentatively assigned to Fe-containing aggregates of triple-positive charge. This conforms with the possible enrichment of successive aggregates with an additional H_3_PG molecule (molecular mass of 126 D). The above observations indicate the notable tendency for the formation of the aggregates between [M(CN)_6_]^3−^ anions and H_3_PG in the gas phase, which is in line with the structural data.

### 2.4. DFT Calculations

To shed some light on the strength and nature of the interaction between the [M(CN)_6_]^3−^ anion and H_3_PG molecule(s) in **1**–**3**, dispersion-corrected density functional theory (DFT + D4) [63] calculations were performed employing molecular cluster models, {[M(CN)_6_]^3−^;(**H_2_**PGH)_2_(**H**PGH_2_)_2_}, {[M(CN)_6_]^3−^;**H_2_**PGH} and {[M(CN)_6_]^3−^;**H**PGH_2_}, extracted from the corresponding crystal structures. For a comparison, analogous analyses were also carried out for 4,4′-bipyridyl/H_3_PG {4,4′bpy;**H**PGH_2_} and *trans*-1,2-bis(4-pyridyl)ethylene/H_3_PG {dpe;**H**PGH_2_} clusters extracted from crystal structures reported in Refs. [25,64], respectively. A full description of computational details used in these studies, along with additional calculated results, are provided in the Supplementary Materials (Table S4, Figures S14–S17).

Table 2 and Table S4 list values of interaction energy $\Delta E_{int}$ between the [M(CN)_6_]^3−^ and neighboring H_3_PG molecule(s) (either four following the *cis*-bis(chelated) {[M(CN)_6_]^3−^;(**H_2_**PGH)_2_(**H**PGH_2_)_2_} structural fragment or one corresponding to the double hydrogen-bonded {[M(CN)_6_]^3−^;**H_2_**PGH} and single hydrogen-bonded {[M(CN)_6_]^3−^;**H**PGH_2_} motifs) and between 4,4′bpy or dpe and H_3_PG in {4,4′bpy;**H**PGH_2_} and {dpe;**H**PGH_2_} used as a reference. As can be seen, $\Delta E_{int}$ computed for a given motif does not show a strong dependence on the basis set nor the density functional employed in the calculations, although the double-hybrid functionals (expected to give the most accurate results [65]) systematically indicate somewhat stronger interactions between hydrogen-bond acceptor and hydrogen-bond donor molecules in the examined clusters compared to standard gradient and global hybrid functionals. Note also that tremendously decreased magnitude interaction energies were obtained for the motifs extracted from **1**–**3** when the acetonitrile continuum solvent model was employed in the calculations (see Table S4), in line with additional energetic stabilization of the charged [M(CN)_6_]^3−^ fragment in such electrostatic medium; as in the crystal structures, the charge of the hexacyanidometallate anion is also screened to some extent by the surrounding moieties (cations in particular), and we expect the [M(CN)_6_]^3−^/H_3_PG interaction energies to be smaller in magnitude than those determined by the gas-phase calculations although definitely not so diminished as indicated by solvation ones. Nevertheless, all the methods uniformly demonstrate: (i) a slight increase in the magnitude of interaction energies between [M(CN)_6_]^3−^ and H_3_PG molecule(s) in the *cis*-bis(chelated) {[M(CN)_6_]^3−^;(**H_2_**PGH)_2_(**H**PGH_2_)_2_} and double hydrogen-bonded {[M(CN)_6_]^3−^;**H_2_**PGH} when going from system **1** (M = Cr) through **3** (Co) to **2** (Fe), (ii) overall similar values of the interaction energy between [M(CN)_6_]^3−^ and H_3_PG in the single hydrogen-bonded {[M(CN)_6_]^3−^;**H**PGH_2_} motif in all three compounds, and (iii) the pronounced (more than double) increase in the magnitude of these energies when compared with the corresponding single hydrogen-bonded reference clusters. We also note in passing that the computed interaction energies for {[M(CN)_6_]^3−^;**H_2_**PGH} and {[M(CN)_6_]^3−^;**H**PGH_2_} overall do not appear to be additive, that is, their respective sum does not reproduce (in fact, it generally exceeds) the value obtained for {[M(CN)_6_]^3−^;(**H_2_**PGH)_2_(**H**PGH_2_)_2_}. This is not surprising as, in such 1:1 motifs, the H-bonding component of the [M(CN)_6_]^3−^/H_3_PG interaction might be more effective compared to that in the motif with [M(CN)_6_]^3−^ surrounded by four hydrogen-bond donor molecules. Even the interaction energy in the double {[M(CN)_6_]^3−^;**H_2_**PGH} synthon is overall slightly smaller (less negative) than the sum of interaction energies of two simple {[M(CN)_6_]^3−^;**H**PGH_2_} motifs. This small energetical penalty might be attributed to a minor steric hindrance that appears when two resorcinol-like H-O-ring groups accommodate the *cis*-oriented cyanido ligands in [M(CN)_6_]^3−^, which is in agreement with the hydrogen-bond distances observed for both motifs in the crystal structure. Nevertheless, this does not diminish the overall strength and significance of the interaction within the double cyclic synthons. 

**Figure 5:**
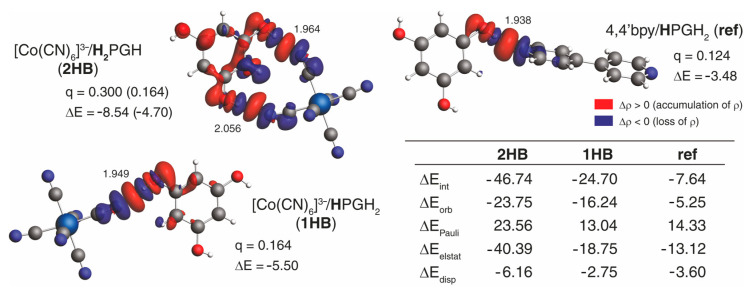
Results of ETS-NOCV analysis of the interaction between [M(CN)_6_]^3−^ anion and H_3_PG molecule in {[Co(CN)_6_]^3−^;**H_2_**PGH} and {[Co(CN)_6_]^3−^;**H**PGH_2_} molecular clusters extracted from the crystal structure of **3** and between 4,4′-bipyridyl (4,4′bpy) and H_3_PG in {4,4′bpy;**H**PGH_2_} molecular cluster extracted from the crystal structure reported in Ref. [64] used here as a reference. Isosurfaces (±0.0005 au) of dominant NOCV contributions to the differential electron density Δρ describing hydrogen bonding along with their charge (q in e) and orbital energy (ΔE in kcal mol^−1^) assessment. Two values of q and ΔE provided for the {[Co(CN)_6_]^3−^;**H_2_**PGH} motif correspond to the total assessment for both hydrogen bonds and to the shortest one only (given in parentheses). Numbers listed close to the O–H···N contacts are the hydrogen-bond distances, in Å. In the table: The corresponding interaction energy components (in kcal mol^−1^) as obtained using the ETS energy decomposition scheme are presented. Based on BLYP + D4//TZP calculations.

To comment on the nature of [M(CN)_6_]^3−^/H_3_PG interaction in **1**–**3**, extended transition state–natural orbitals for chemical valence (ETS-NOCV) [66] charge and bonding-energy decomposition analyses were then performed for double hydrogen-bonded {[Co(CN)_6_]^3−^;**H_2_**PGH} and single hydrogen-bonded {[Co(CN)_6_]^3−^;**H**PGH_2_} motifs, the results of which, along with those obtained for the reference systems, are presented in Figure 5 and Figure S15–S17. As expected, the dominant NOCV contributions to the differential electron density, that is, the redistribution of electron density around [Co(CN)_6_]^3−^, 4,4′bpy or dpe and H_3_PG in the considered molecular clusters, describe hydrogen-bonding interactions and clearly show its covalent nature via visible hydrogen-bond acceptor and donor charge-transfer (CT) interaction between the occupied lone-pair of nitrogen and the unoccupied σ* orbital of the O–H bond, accompanied in the case of [Co(CN)_6_]^3−^/H_3_PG by the participation of π-type orbitals of both moieties (see below) [66]. The orbital component of the hydrogen bonds present in {[Co(CN)_6_]^3−^;**H_2_**PGH} and {[Co(CN)_6_]^3−^;**H**PGH_2_} appears to be rather energetically similar with a slight enhancement as that in {[Co(CN)_6_]^3−^;**H**PGH_2_}, in line with its shortest distance. The one observed in {4,4′bpy;**H**PGH_2_}, of comparable hydrogen-bonding separation as in {[Co(CN)_6_]^3−^;**H**PGH_2_}, demonstrates a non-negligible decrease, indicating that nitrogen in negatively charged [M(CN)_6_]^3−^ might be a better hydrogen-bond acceptor. The analysis of the interaction energy components obtained using the ETS energy decomposition scheme (see Figure 5 and Figure S15–S17) enabled us to elucidate further a large difference in the interaction between [Co(CN)_6_]^3−^ and H_3_PG and between 4,4′bpy or dpe and H_3_PG in the considered molecular clusters. Namely, the results show that the strong interaction in {[Co(CN)_6_]^3−^;**H_2_**PGH} and {[Co(CN)_6_]^3−^;**H**PGH_2_} is determined by both electrostatic $\Delta E_{elstat}$ and orbital-interaction $\Delta E_{orb}$ components [67], with the latter being represented not only by the σ-CT hydrogen-bonding channel but primarily, as indicated by the analysis of other energetically relevant NOCV contributions, by the polarization (intra-CT) of the π-electron system within H_3_PG, enhanced likely due to ion–dipole interaction imposed by the negative charge of the [Co(CN)_6_]^3−^. Such electron-transfer channels in {4,4′bpy;**H**PGH_2_} and {dpe;**H**PGH_2_} are visibly diminished, which seems to be directly responsible for a pronounced decrease in the magnitude of the orbital-interaction contribution (even for {dpe;**H**PGH_2_}, for which a shorter hydrogen-bond distance significantly strengthens its corresponding orbital component, see Figure S17). This decrease in $\Delta E_{orb}$ along with less stabilizing $\Delta E_{elstat}$ provides less counterbalance for the repulsive Pauli interaction and, accordingly, a significant drop in the absolute values of $\Delta E_{int}$ for {4,4′bpy;**H**PGH_2_} and {dpe;**H**PGH_2_} was observed. 

### 2.5. Spectroscopic Studies

#### 2.5.1. Vibrational Studies 

**Figure 6:**
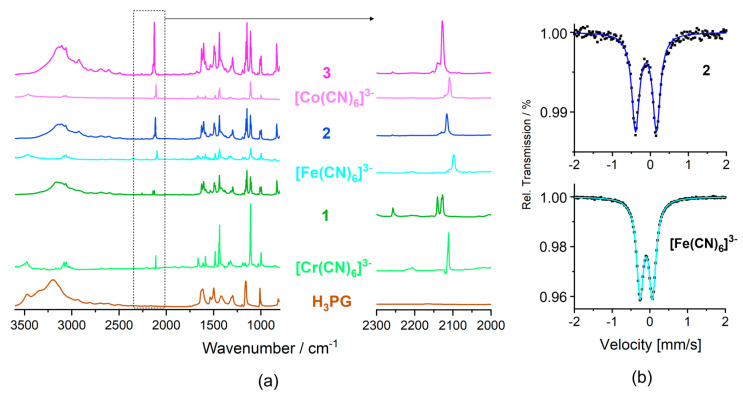
(**a**) Infrared spectra of **1**-**3** in the absorption mode compared with the spectra of H_3_PG and (PPh_4_)_3_[M(CN)_6_]·*n*H_2_O precursor salts with enlargement of including the *ν*(C≡N) stretching vibration range shown on the right. (**b**) ^57^Fe Mössbauer spectra of **2** (**top**) and of the (PPh_4_)_3_[Fe(CN)_6_]·7H_2_O precursor (**bottom**).

The IR spectra of **1**–**3** in the 4000–700 cm^−1^ and 600–100 cm^−1^ range (Figure 6a, Figures S18 and S19) combine the spectral features of all relevant structural components: *ν*(O-H) vibrations of H_3_PG; *ν*(C-H) vibrations of H_3_PG, PPh_4_^+^ and MeCN; *ν*(C≡N) vibrations of [M(CN)_6_]^3−^ and MeCN, and a full set of skeletal vibrations of H_3_PG and PPh_4_^+^ (*for details see* Materials and methods). H_3_PG and all co-crystal salts **1**–**3** show a significant spread of the band assigned to the *ν*(O-H) vibrations in the 3600–2500 cm^−1^ range due to the presence of a hydrogen-bond network. However, for **1–3,** the high wavenumber limit and the position of the band maximum are visibly shifted to lower energy (all in cm^−1^): from 3600 to ca. 3470 and from 3195 to ca. 3120, respectively. This can be interpreted in terms of the increased strength of hydrogen bonds (and decreased strength of O-H bonds) in **1**–**3** compared to those in H_3_PG [35,36,68], expected based on the difference in Brønsted acidity of H_3_PG and H_2_O and the basicity of H_3_PG and [M(CN)_6_]^3−^. The bands characteristic of *ν*(C≡N) are notably shifted to the higher wavenumber (*ca.* 20 cm^−1^) in **1**–**3** compared to the precursors (all in cm^−1^): from 2112 m towards 2126 m, 2129 m, and 2140 m for **1**, from 2098 s, 2109 w, and 2116 vw towards 2116 s, 2129 w and 2135 vw for **2**, and from 2106 m(sh), 2109 s and 2122 towards 2128 s and 2140 m for **3** (Figure 6a) [20,69,70,71]. The above spectral changes are consistent with the occurrence of CN^−^⋅⋅⋅H-O_H3PG_ hydrogen bonds, notably stronger and extended, compared to rather local CN^−^⋅⋅⋅H-O_H2O_ hydrogen bonds observed in the crystal structures of (PPh_4_)_3_[M(CN)_6_]·*n*H_2_O salts (Figure S5) [58,59]. While the fingerprint region is not diagnostic for the modification of the vibrational (and electronic) structure (Figure S18), the spectra in the FIR region 600–100 cm^−1^ indicate notable hipsochromic shifts of the skeletal δ(Cr-C-N), δ(Cr-C-N), and ν(Cr-C) vibrations, and bathochromic shifts of selected H_3_PG vibrations (Figure S19), being representative to the whole **1**–**3** series [9,69,72]. Modification of vibrational structure in **1** compared to the precursors, occurring due to a relocation of the electronic density as the result of the hydrogen-bonding architecture, was also confirmed by Raman spectra (Figures S20–S22) [69]. 

#### 2.5.2. ^57^Fe Mössbauer Spectra

The solid-state ^57^Fe Mössbauer spectra for **2** and the (PPh_4_)_3_[Fe(CN)_6_]·7H_2_O reference are presented in Figure 6b. Both spectra were reproduced using single doublets assignable to the low-spin (LS) Fe^III^ state expected for the LS [Fe(CN)_6_]^3−^ anion. Co-crystal salt **2** reveals the isomeric shift δ**_2_** = –0.11 mm s^−1^, and quadrupole splitting QS**_2_** = 0.55 mm s^−1^. δ**_2_** is smaller than δ**_ref_** = –0.09 mm s^−1^ for the reference salt, which is in line with more significant electron density removal from the 3 d metal valence orbitals expected for more extended and stronger hydrogen bonds _CN_N⋅⋅⋅H-O_H3PG_ in **2** compared to those present in the reference salt. QS**_2_** is considerably larger than QS**_ref_** = 0.33 mm s^−1^ for the reference, which might be related to a specific distribution of the cyanido ligands of the local C_2_ symmetry and slightly decreased degeneracy of the t_2g_ orbital set due to the bis(chelate)-like arrangement of four H_3_PG hydrogen bond donors. The observed negative correlation between δ and QS change is in line with the tendency found for a set of hydrated and dehydrated PBAs [1,73].

#### 2.5.3. UV-Vis Electronic Absorption Spectra 

**Figure 7:**
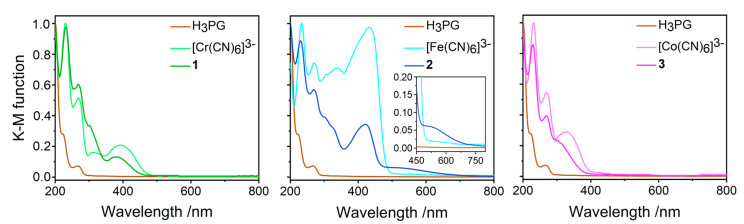
Electronic absorption UV-Vis spectra of **1**–**3** in the solid-state compared to H_3_PG and respective (PPh_4_)_3_[M(CN)_8_]⋅nH_2_O precursors. The reflectance spectra were recalculated into the Kubelka-Munk finction. Colors: **1**—green, **2**—blue, and **3**—pink.

The colors of the starting materials are: white for H_3_PG, pale yellow for [Cr(CN)_6_]^3−^ salt, yellow for [Fe(CN)_6_]^3−^ salt, and white for [Co(CN)_6_]^3−^ salt, whereas the obtained co-crystal salts are yellow (**1**), yellow–brownish (**2**), and pale yellow (**3**). The solid-state UV-Vis absorption spectra for **1–3,** together with the spectra of H_3_PG and of the respective (PPh_4_)_3_[M(CN)_6_]·*n*H_2_O precursors in the 200–800 nm range, are presented in Figure 7. In general, the most important spectral features characteristic for [M(CN)_6_]^3−^ and H_3_PG units were reproduced for **1**–**3**, and below, we only discuss the directly detectable changes observed individually for each product compared to the spectra of the precursors.

Compounds **1** and **3** showed the hypsochromic shift of the bands assignable to the lowest energy spin-allowed transitions as compared to (PPh_4_)_3_[Cr(CN)_6_]·2H_2_O and (PPh_4_)_3_[Co(CN)_6_]·6H_2_O, respectively. For **1**, the energies of the ^4^A_2g_ → ^4^T_2g_ (^4^F) and ^4^A_2g_ → ^4^T_1g_ (^4^F) transitions [9,69,74,75,76,77] were increased from 388 to 380 nm (ΔE = 550 cm^−1^) and from 315 to ca. 290 nm (ΔE of at least of 2700 cm^−1^; this estimation lacks exactness due to the bands’ overlap). For **3**, the energy of the ^1^A_1g_ → ^1^T_1g_ transition [70,76,77,78,79] was shifted from 324 to ca. 297 nm (Δ of at least 2800 cm^−1^). For the Fe(CN)_6_]^3−^ analogue, the lowest-energy range was dominated by ligand-to-metal charge-transfer (LMCT) σ(CN^–^) → π(2t_2g_) transitions with a possible admixture of one of the ligand-field (LF) spin-forbidden transitions [71,76,77,80]. While the whole band was also shifted to a higher energy from 433 nm for the reference towards 422 nm for **2** (ΔE = 600 cm^−1^), another feature centered at ca. 490 nm appeared for **2**, covering the range up to 750 nm. All observed changes should be interpreted in terms of the stabilization or destabilization of the relevant metal and cyano-ligand orbitals involved in the transitions due to the relocation of electronic density along the σ- and π-channels under the impact of the electrostatic field provided by the species surrounding cyanido-ligands. For **1** and **3**, the relative increase of Δ_O_ splitting might be directly inferred due to the stronger electrostatic field imposed by six CN^−^⋅⋅⋅H-O_H3PG_ hydrogen bonds, compared to the CN^−^⋅⋅⋅H-O_water_ hydrogen bonds observed in the crystal structures of (PPh_4_)_3_[M(CN)_6_]·*n*H_2_O salts [58,59]. The range of the Δ_O_ change is comparable to those reported for [Cr(CN)_6_]^3−^ and [Co(CN)_6_]^3−^ anions in the solid matrices of alkali metal halides; however, a sign of this change might depend on the distance and geometry of CN⋅⋅⋅cation motif [69,70,75]. The theoretical consideration of the energy levels for the mono-ionized [Co(CN)_6_]^4–^ anion suggests that our observations for **1** and **3** might be due to the relative stabilization of 2t_2g_ orbitals, from where the electrons are excited in the LF states [79]. In the case of **2,** the interpretation is not so straightforward as the 2t_2g_ orbitals (the incomplete configuration 2t_2g_^5^) involved in the LMCT transitions are the electron recipient levels. Thus, in this case, one should also consider the relocation of electronic density on the relevant lower energy orbitals (of 3t_1u_ and/or 2a_1g_ type) or some splitting of the involved orbitally degenerated states under the electrostatic field of hydrogen bonds [77]. The low energy spin forbidden bands of [Cr(CN)_6_]^3−^ and [Co(CN)_6_]^3−^ were scarcely detectable in our setup and were not considered in the analysis. A more precise description of the electronic structure of our co-crystal salts might be obtained with the application of more advanced experimental methods based on X-ray absorption and emission, or ultrafast photoelectron spectroscopy coupled with transient infrared studies combined with modern computational methods [71,78,80,81,82,83].

#### 2.5.4. Photoluminescence Studies

**Figure 8:**
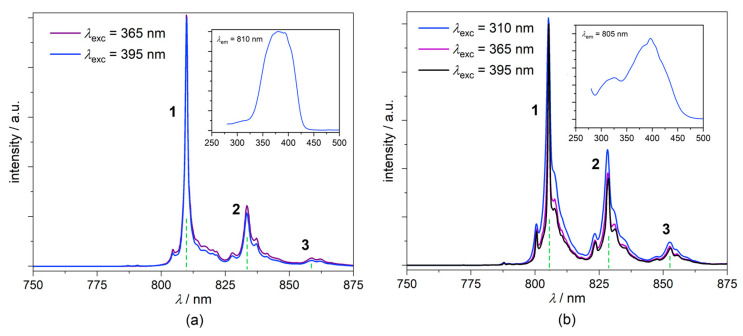
^2^E_g_ → ^4^A_2g_ emission spectra of **1** (**a**) and (PPh_4_)_3_[Cr(CN)_6_]⋅2H_2_O reference (**b**) at various excitation wavelengths in *T* = 77 K. The insets show the excitation spectra at *λ*_em_ related to the highest emission intensity in emission spectra.

The photoluminescence spectra of **1** and the (PPh_4_)_3_[Cr(CN)_6_]⋅2H_2_O reference at 77 K are presented in Figure 8, both revealing three distinguishable bands of the location and maximum lines specified in Table 3. The photoluminescence pattern observed in the range 750–875 nm is assigned to the ^2^E_g_ → ^4^A_2g_ phosphorescence characteristic of various inorganic solids and hybrid molecular solids involving [Cr(CN)_6_]^3−^ moiety [9,69,75,84,85] and may be interpreted in terms of its vibronic properties. The positions of these bands are usually indicated in respect to the R_1_ (0′-0) emission line (here not measured, however, expected to be located at ca. 795–800 nm) and might be attributed to the specific fundamental modes involving some of the skeletal δ(C-Cr-C), δ(Cr-C-N) and δ(Cr-C) vibrations (below 450–500 cm^−1^; ν_9_, ν_13_, ν_7_, ν_8_, and ν_12_ in the increasing energy order) and combination modes (above 500 cm^−1^) [9,69,75]. The systematic bathochromic shift of ca. 5 nm was observed, going from (PPh_4_)_3_[Cr(CN)_6_]⋅2H_2_O to **1**, which might be interpreted in terms of the modification of molecular surroundings described above, and correlates with the hypsochromic shift of the ^4^A_2g_ → ^4^T_2g_ (^4^F) and ^4^A_2g_ → ^4^T_1g_ (^4^F) transitions in the UV-Vis spectra. This nicely corresponds with the systematic increase of the relevant absorption energy and decrease of the ^2^E_g_ → ^4^A_2g_ phosphorescence energy observed for the solid phases of (PPh_4_)_3_[Cr(CN)_6_]⋅2H_2_O, **1**, and K_3_[Cr(CN)_6_], coming from the first one to the last one [69,75,84]. The emission lifetimes *τ*_1_ determined using the equation corresponding to the single decay process are (all in ms): 6.8 (77 K) and 5.5 (298 K) for (PPh_4_)_3_[Cr(CN)_6_]⋅2H_2_O, and 5.1 (77 K) and 4.5 (298 K) for **1** (*λ*_exc_ = 395 nm followed at the various accessible emission lines) (Figures S23 and S24). The observed slight decrease of *τ*_1_ for **1** might be tentatively attributed to the specific character of hydrogen bond architecture in **1** described above. The shortening of lifetimes coincides with the decrease of average luminescence quantum yields measured at room temperature, from 10.9(2)% for (PPh_4_)_3_[Cr(CN)_6_]⋅2H_2_O to 8.84(4)% for **1**. More detailed information could be inferred from LHe measurements, supported by computational methods, which are beyond the scope of this study.

## 3. Conclusions and Perspectives

The PPh_4_^+^ cation-assisted supramolecular self-assembly between the [M(CN)_6_]^3−^ and triple-point H_3_PG hydrogen bond donor led to the formation of the new 2D hydrogen-bonded network {[M(CN)_6_]^3−^;(H_3_PG)_2_} of square-like topology alternative to the 2D hexagonal networks formed between H_3_PG and [Co_2_Fe_2_(CN)_6_(tp*)_2_(bpy*)_4_]^2+^ [23] or between [M(CN)_6_]^3−^ and bisaminidnium dications [13]. [M(CN)_6_]^3−^ complexes located in the nodes of this network are surrounded by four H_3_PG molecules that form two single M-CN⋅⋅⋅HO-Ar and two double cyclic M(-CN⋅⋅⋅HO-)_2_Ar synthons into an original non-covalent *cis*-bis(chelated) {[M(CN)_6_]^3−^;(**H_2_**PGH)_2_(**H**PGH_2_)_2_} motif, recognized by analogy with the *cis*-bis(chelated) 6-coordinate [ML_2_A_2_] complexes. Hirshfeld analysis indicated the notable intensity of the underlaying interactions. The strong mutual affinity of both tectons was confirmed by ESI-MS spectrometry and was nicely quantified by the substantial energies of interactions reaching ca. 23–27 kcal mol^−1^ for simple {[M(CN)_6_]^3−^;**H**PGH_2_} and ca. 45–50 kcal mol^−1^ for double cyclic {[M(CN)_6_]^3−^;**H_2_**PGH} motifs obtained based on the gas-phase DFT + D4 calculations for the molecular clusters extracted from the crystal structures. Thus, the H_3_PG proved to be an efficient double-point receptor unit for [M(CN)_6_]^3−^ anions thanks to the molecular matching between the resorcinol-like face of H_3_PG and *cis*-oriented pairs of cyanido ligands of [M(CN)_6_]^3−^. The observed {[M(CN)_6_]^3−^;(**H_2_**PGH)_2_(**H**PGH_2_)_2_} motif revealed chiral (C_2_) local surroundings of the [M(CN)_6_]^3−^ complex, which might be of importance in the broader context of supramolecular design towards interesting symmetry-related structure-properties schemes. The spectroscopic characteristics of **1**–**3** revealed notable modifications as compared to the (PPh_4_)_3_[M(CN)_6_]⋅*n*H_2_O references characterized by less saturated hydrogen-bond surroundings of [M(CN)_6_]^3−^ involving relatively weaker hydrogen-bond donors; this also includes the photoluminescent properties of the [Cr(CN)_6_]^3−^ anion. 

The above findings might be important from the standpoint of the design of modular multisite anion receptors dedicated to binding of d-metallates and the development of alternative pathways towards the controlled synthesis of new multicomponent (coordination-based, hybrid organic-inorganic, etc.) architectures and materials of functional features. In particular, the targeted CCDC search indicates the existence of several very interesting complex molecular motifs exhibiting multiple resorcinol groups [86,87,88,89,90,91,92,93]; they may be definitely considered as potential platforms for multisite anions receptors exploiting the synthons described in this manuscript. Advanced research in this direction is underway in our group. 

## 4. Materials and Methods

K_3_[Fe(CN)_6_], K_3_[Co(CN)_6_], K_3_[Cr(CN)_6_], PPh_4_Cl, PPh_4_Br, 1,3,5-trihydroxybenzene, and solvents were purchased from a commercial source (Sigma-Aldrich, Alfa Aesar, etc.) and used without further purification.

### 4.1. Synthetic Procedures

(PPh_4_)_3_[Fe(CN)_6_]·7H_2_O, (PPh_4_)_3_[Co(CN)_6_]·6H_2_O, and (PPh_4_)_3_[Cr(CN)_6_]·2H_2_O were prepared by metathesis of the corresponding potassium salts of the complexes with PPh_4_Cl.

**Synthesis of 1.** Acetonitrile solutions of (PPh_4_)_3_[Cr(CN)_6_]·2H_2_O (0.227 g, 0.18 mmol in 15 mL of CH_3_CN) and H_3_PG (0.0324 g, 0.2 mmol in 15 mL of CH_3_CN) were mixed to obtain a colorless solution, and the mixture was tightly closed in the vessel. After one day, pale yellow crystals of **1** appeared. The crystals were filtered and washed with cold acetonitrile (10 mL, 2 °C) and dried in air. The composition of (PPh_4_)_3_[Cr(CN)_6_](H_3_PG)_2_·2MeCN was defined by a single-crystal X-ray diffraction analysis. Phase purity was proved by XRD data. Yield: 0.168 g, 60.0%. Elemental analysis. Calc. for C_94_H_78_CrN_8_O_6_P_3_ (Mw = 1560.55 g·mol^−1^): C, 72.34%; H, 5.04%; N, 7.18%. Found: C, 72.1%; H, 5.0%; N, 7.1%. IR (KBr, cm^−1^): 3470–2500 *v*(O-H), 3059 *v*(C-H), 2257 *v*(C≡N) (MeCN), 2126, 2129, 2140 *v*(C≡N) ([Cr(CN)_6_]^3−^), other: 1585, 1481, 1438, 1186, 1109, and 995 (vibrations of PPh_4_^+^) 1626, 1606, 1491, 1419 br, 1297, 1155,1146, 1010, 1004, and 832 (vibrations of H_3_PG). Solubility: MeOH–good, CH_3_CN–poor. Stability: composition stable up to ca. 80 °C, then loses 2 crystallization MeCN molecules in the range 80–140 °C; above 190 °C massive decomposition occurs (Figure S25).

**Synthesis of 2.** Acetonitrile solutions of (PPh_4_)_3_[Fe(CN)_6_]·6H_2_O (0.240 g, 0.018 mmol in 15 mL of CH_3_CN) and H_3_PG (0.0324 g, 0.2 mmol in 15 mL of CH_3_CN) were mixed to obtain a pale yellow solution, and the mixture was tightly closed in the vessel. After one day, brown–yellow crystals of **2** appeared. The crystals were filtered and washed with cold acetonitrile (10 mL, 2 °C) and dried in air. The composition of (PPh_4_)_3_[Fe(CN)_6_](H_3_PG)_2_·2MeCN was defined by a single-crystal X-ray diffraction analysis. Phase purity was proved by XRD data. Yield: 0.180 g, 64.0%. Elemental analysis. Calc. for C_94_H_78_FeN_8_O_6_P_3_ (Mw = 1564.40 g·mol^−1^): C, 72.17%; H, 5.03%; N, 7.16%. Found: C, 72.2%; H, 5.0%; N, 7.2%. IR (KBr, cm^−1^): 3445–2500 *v*(O-H), 3063 *v*(C-H), 2257 *v*(C≡N) (MeCN), 2116, 2130, 1441 *v*(C≡N) ([Fe(CN)_6_]^3−^); other: 1585, 1481, 1438, 1186, 1109, and 995 (vibrations of PPh_4_^+^) 1626, 1606, 1491, 1419 br, 1297, 1155,1146, 1010, 1004, 832 (vibrations of H_3_PG). Solubility: MeOH–good, CH_3_CN–poor. Stability: composition stable up to ca. 70 °C, then loses 2 crystallization MeCN molecules in the range 70–130 °C; above 170 °C massive decomposition occurs (Figure S25).

**Synthesis of 3.** Acetonitrile solutions of (PPh_4_)_3_[Co(CN)_6_]·7H_2_O (0.244 g, 0.018 mmol in 15 mL of CH_3_CN) and H_3_PG (0.0324 g, 0.2 mmol in 15 mL of CH_3_CN) were mixed to obtain a colorless solution immediately, and the mixture was tightly closed in the vessel. After one day, colorless crystals of **3** appeared. The crystals were filtered and washed with cold acetonitrile (10 mL, 2 °C) and dried in air. The composition of (PPh_4_)_3_[Co(CN)_6_](H_3_PG)_2_·2MeCN was defined by a single-crystal X-ray diffraction analysis. Phase purity was proved by XRD data. Yield: 0.126 mg, 44.8%. Elemental analysis. Calc. for C_94_H_78_CoN_8_O_6_P_3_ (Mw = 1567.48 g·mol^−1^): C, 72.02%; H, 5.02%; N, 7.15%. Found: C, 72.12%; H, 4.9%; N, 7.1%. IR (KBr, cm^−1^): 3460–2500 *v*(O-H), 3066 *v*(C-H), 2257 *v*(C≡N) (MeCN), 2128, 2140 *v*(C≡N) ([Co(CN)_6_]^3−^), other: 1585, 1481, 1438, 1186, 1109, 995 (vibrations of PPh_4_^+^) 1626, 1606, 1491, 1419 br, 1297, 1155,1146, 1010, 1004, 832 (vibrations of H_3_PG). Solubility: MeOH–good, CH_3_CN–poor. Stability: composition stable up to ca. 70 °C, then loses 2 crystallization MeCN molecules in the range 70–120 °C; above 230 °C massive decomposition occurs (Figure S25).

### 4.2. X-ray Diffraction Analysis

Single crystal X-ray diffraction data for all compounds were collected using a Bruker D8 Quest Eco diffractometer equipped with a Photon II detector and a Mo Kα (λ = 0.71073 Å) radiation source with a graphite monochromator and Oxford Cryostream cooling system. Crystals for measurement were taken from the mother solution and covered by NVH immersion oil. All measurements were performed in 100.0 K. Data reduction and cell parameter refinement were performed using Apex software with included SAINT and SADABS programs. Intensities of reflections for the sample absorption were corrected using the multiscan method. Structures were solved by the intrinsic phasing method and refined anisotropically with weighted full-matrix least-squares on F^2^ using SHELXT [94] and SHELXL [95] programs with Olex 2 graphic interface [96]. 

Hydrogen atoms within structures were placed in idealized positions and refined using a riding coordinate model with isotropic displacement parameter set at 1.2–1.5 times U_eq_ of appropriate carrier atoms. Crystal data and structure refinement parameters are summarized in Table S1. The structural figures in the article were prepared using the latest Mercury software [97]. The crystal structures are deposited in the CCDC database. The deposition numbers are 2,175,600 (**1**), 2,175,601 (**2**), and 2,175,602 (**3**).

### 4.3. Physical Techniques and Calculations

Elemental analyses of CHNS were performed on the air-dried samples using the Elemental Vario Micro Cube CHNS analyzer. Powder X-ray diffraction patterns for **1**, **2**, and **3** in 0.5 mm glass capillary were collected on a D8 Advance Eco (Bruker) using a Cu−Kα radiation source. The thermogravimetric (TGA) curves for the polycrystalline samples were collected using TG209 F1 Libra thermogravimetric analyzer with aluminum pans as holders. The data were collected in the temperature range of 21–400 °C under a nitrogen atmosphere with a heating rate of 1 °C per minute. Infrared (IR) absorption spectra in the range 4000–675 cm^−1^ were measured on the selected single-crystals using a Nicolet iN10 MX Fourier transform infrared microscope. Far infrared (FIR) spectra in the range 600–100 cm^−1^ were measured on the powder samples dispersed Apiezon N grease using an FT-IR Bruker Vertex 70 V spectrometer. Raman spectra in the range 3200–100 cm^−1^ were recorded on the microcrystalline samples with a Renishaw inVia Raman spectrometer with the excitation line 514.5 nm of Ar laser. The transmission ^57^Fe Mössbauer spectra were collected in 1024 channels, with a 10 mCi ^57^Co source in an Rh matrix, at room temperature using a Wissel spectrometer. The velocity scale was calibrated using the α-Fe foil standard. The powder sample was directly placed in copper rings and sealed with Kapton foil. The background spectra of the sample holders did not reveal any significant contribution to the main spectra. Mössbauer spectra were fitted with the use of the WinNormos-for-Igor software package, assuming the Lorentzian shape of the resonance lines, i.e., the saturation effects were not included. For each compound, one quadrupole doublet was considered in the fitted model, assigned to the Fe^III^ (LS) electron state. Diffuse reflectance spectra in the UV-Vis-NIR range were performed for the ground powder samples mixed with BaSO_4_ (2 mass %) using a Shimadzu UV-3600i Plus spectrophotometer equipped with the 50 mm integrating sphere. The spectra were recalculated according to the Kubelka–Munk equation. Solid-state photoluminescent characterization for all reported compounds was performed using an FS5 spectrofluorometer (Edinburgh Instruments) equipped with a Xe arc lamp (150 W, excitation spectra) or a 365 nm diode flashlight (10 W, emission spectra) serving as excitation sources, and a Hamamatsu photomultiplier of the R928P type as a detector. All lifetime measurements were conducted using the same spectrofluorometer employing a multichannel scaling module with a microsecond Xe flashlamp (5 W), while the collected data curves were fitted using a monoexponential decay function within a Fluoracle software (Edinburgh Instruments). Absolute quantum yields (QYs) were measured by a direct excitation method using the Xe arc lamp, an integrating sphere module (SC-30), and a Teflon pin as a reference, while the related calculations were performed within the implemented software. The Fluoracle program was also employed for the background corrections and a smoothing procedure, while for the data collected using the 365 nm flashlight, the application of a non-linear baseline was determined using the Asymmetric Least-Square Smoothing method (OriginPro 2021b) was found necessary. 

### 4.4. Hirshfeld Analysis 

The structural interaction analyses based on Hirshfeld surfaces were performed in CrystalExplorer [60,61,62].

### 4.5. Quantum-Chemical Calculations and Analyses

All computations were performed at the density functional theory (DFT) level employing molecular cluster models extracted from the corresponding crystal structures. A full description of computational details used in these studies is provided in the Supplementary Materials [63,66,98,99,100,101,102,103,104,105,106,107,108,109].

## Figures and Tables

**Table 1:** The most important hydrogen-bonding parameters and related angles in **1**–**3** (in [Å] or in [deg]; see also Figure 1c).

Compound	1	2	3
Formula	double hydrogen bonds		
N2⋅⋅⋅O2#1 *^a^*	2.769	2.777	2.787
N2⋅⋅⋅H2#1	1.959	1.940	1.964
N2⋅⋅⋅H2#1-O2#1	176.89	173.77	178.21
N3⋅⋅⋅O3#1	2.815	2.827	2.835
N3⋅⋅⋅H3#1	2.016	1.987	2.056
N3⋅⋅⋅H3#1-O3#1	179.53	178.09	177.10
	related angles in [M(CN)_6_]^3−^		
M1-N2-C2	176.70	177.62	177.89
M1-N3-C3	171.31	173.53	173.52
C2-M1-C3	84.87	85.19	85.87
N2⋅⋅⋅M1⋅⋅⋅N3	80.82	82.01	82.75
	single hydrogen bonds		
N1⋅⋅⋅O1	2.733	2.751	2.762
N1⋅⋅⋅H1	1.944	1.916	1.949
N1⋅⋅⋅H1-O1	174.67	172.81	173.99
	related angles in [M(CN)_6_]^3−^		
M1-C1-N1	176.72	177.35	177.63
C1-M1-C *^b^*	88.39	89.24	89.27
	90.18	91.13	91.08
	91.37	91.02	91.38
	88.56	88.55	88.81
N1⋅⋅⋅M1⋅⋅⋅N *^b^*	88.37	89.14	89.19
	90.47	91.47	91.62
	92.26	91.89	92.06
	87.60	87.87	88.00

*^a^* The symmetry operation #1 is −1/2 + *x*,−1/2 + *y*,*z*. *^b^* Close-to-right angles involving M1-C1 bond and M1-C1-N1 angle. Compared to the C2-M1-C3 and N2⋅⋅⋅M1⋅⋅⋅N3 angles.

**Table 2:** DFT-computed interaction energy $\Delta E_{int}$ values (in kcal mol^−1^) between [M(CN)_6_]^3−^ (M = Cr, Fe, Co) anion and H_3_PG molecule(s) in molecular clusters {[M(CN)_6_]^3−^;(**H_2_**PGH)_2_(**H**PGH_2_)_2_}, {[M(CN)_6_]^3−^;**H_2_**PGH}, and {[M(CN)_6_]^3−^;**H**PGH_2_} extracted from the crystal structures of **1**, **2**, and **3**. For comparison, the corresponding interaction energies between 4,4′-bipyridyl (4,4′bpy) and H_3_PG in molecular cluster {4,4′bpy;**H**PGH_2_} extracted from the crystal structure reported in Ref. [64] are listed.

	[Cr(CN)_6_]^3−^	[Fe(CN)_6_]^3−^	[Co(CN)_6_]^3−^	4,4′bpy
BLYP + D4//TZP *^a^*				
(**H_2_**PGH)_2_ (**H**PGH_2_)_2_	−125.33	−133.58	−131.42	–
**H_2_**PGH	−44.98	−48.44	−46.74	–
**H**PGH_2_	†	−27.30	−24.70	−7.64
B3LYP + D4//TZP *^a^*				
(**H_2_**PGH)_2_ (**H**PGH_2_)_2_	−124.87	−133.38	−130.88	–
**H_2_**PGH	−44.65	−47.98	−46.40	–
**H**PGH_2_	−22.30	−24.29	−23.99	−7.70
rev-DOD-BLYP + D4//TZ2P *^a^*				
(**H_2_**PGH)_2_ (**H**PGH_2_)_2_	−134.00	−142.66	−138.84	–
**H_2_**PGH	−47.28	−51.02	−48.77	–
**H**PGH_2_	−23.99	−26.60	−26.46	−9.50

*^a^* The results shown were computed in a vacuum with dispersion-corrected DFT + D4 employing exchange–correlation density functionals belonging to different classes of approximation, BLYP (gradient), B3LYP (global hybrid), and rev-DOD-BLYP (double hybrids), and the indicated basis set (TZP or TZ2P). See Supplementary Materials for a full set of calculated data. † Calculations failed to reach SCF convergence for the motif.

**Table 3:** The energy of ^2^E_g_ → ^4^A_2g_ phosphorescence bands in **1** and (PPh_4_)_3_[Cr(CN)_6_]⋅2H_2_O in 77 K, in nm (cm^−1^).

Compound	1	(PPh_4_)_3_[Cr(CN)_6_]⋅2H_2_O
Band 1 range	802–822 (12,469–12,165)	797–817 (12,547–12,240)
Band 1 max.	810 (12,346)	805 (12,422)
Band 2 range	825–850 (12,121–11,765)	820–842 (12,195–11,876)
Band 2 max.	833 (12,005)	828.5 (12,070)
Band 3 range	855–875 (11,696–11,428)	844–868 (11,848–11,521)
Band 3 max.	858 (11,655)	852.5 (11,730)

## Data Availability

The insight into detailed data might be obtained after the contact with the corresponding author.

## Supplementary Materials

The following supporting information can be downloaded at https://www.mdpi.com/article/10.3390/molecules27134111/s1: additional data for structural description (Figures S1–S5, Tables S1–S3); details of Hirshfeld analysis (Figures S6–S11); additional ESI-MS spectra (Figures S12 and S13); computational methodology description and additional calculated results for molecular clusters in the gas phase, and in continuum solvent model (Figures S14–S17, Table S4); additional IR spectra (fingerprint and FIR region) (Figures S18 and S19); Raman spectra (Figures S20–S22); additional emission spectra and emission lifetimes (Figures S23 and S24); TGA characteristics (Figure S25); related literature citations.

## Sample Availability

Samples of compounds **1**–**3** are available from the authors.
