# Supplementary material for: Supramolecular cis-“Bis(Chelation)” of [M(CN)6]3− (M = CrIII, FeIII, CoIII) by Phloroglucinol (H3PG)
Source: Molecules. 2022 Jun 26;27(13):4111. doi: 10.3390/molecules27134111 (PMC9268030; doi:10.3390/molecules27134111)
Supplement: Supplementary file 1 [file molecules-27-04111-s001.zip › molecules-1774876-supplementary.pdf]

molecules

Supplementary Materials

Supramolecular *cis*-"bis(chelation)" of $[M(CN)_6]^{3-}$ (M = Cr^{III}, Fe^{III}, Co^{III}) by phloroglucinol (H_3PG)

Katarzyna Jędrzejowska[1], Jedrzej Kobylarczyk,[1,2] Dorota Glosz,[1] Emilia Kuzniak-Glanowska[1], Dominika Tabor[1], Monika Srebro-Hooper[1], Jakub J. Zakrzewski[1], Katarzyna Dziedzic-Kocurek[3], Tadeusz M. Muzioł[4], Robert Podgajny[1*]

1 Faculty of Chemistry, Jagiellonian University in Krakow, Gronostajowa 2, 30-387 Kraków, Poland; katarzyna.jedrzejowska@doctoral.uj.edu.pl (K.J.); doglosz@wp.pl (D.G.); kuzniak.emilia@gmail.com (E.K.-G.); dominika.tabor@student.uj.edu.pl (D.T.); monika.srebro@uj.edu.pl (M.S.-H.); jakub.j.zakrzewski@doctoral.uj.edu.pl (J.J.Z.).
2 Institute of Nuclear Physics PAN, Radzikowskiego 152, 31-342 Kraków, Poland; jedrzej.kobylarczyk@ifj.edu.pl (J.K.).
3 Marian Smoluchowski Institute of Physics, Jagiellonian University, Łojasiewicza 11, 30-348 Krakow, Poland; k.dziedzic-kocurek@uj.edu.pl (K.D.-K.).
4 Faculty of Chemistry, Nicolaus Copernicus University in Toruń, Gagarina 7, 87-100 Toruń, Poland; tmuziol@umk.pl (T.M.)
* Correspondence: robert.podgajny@uj.edu.pl. Tel. +48-12-686-2459 (R.P.)

1. Details of structural description

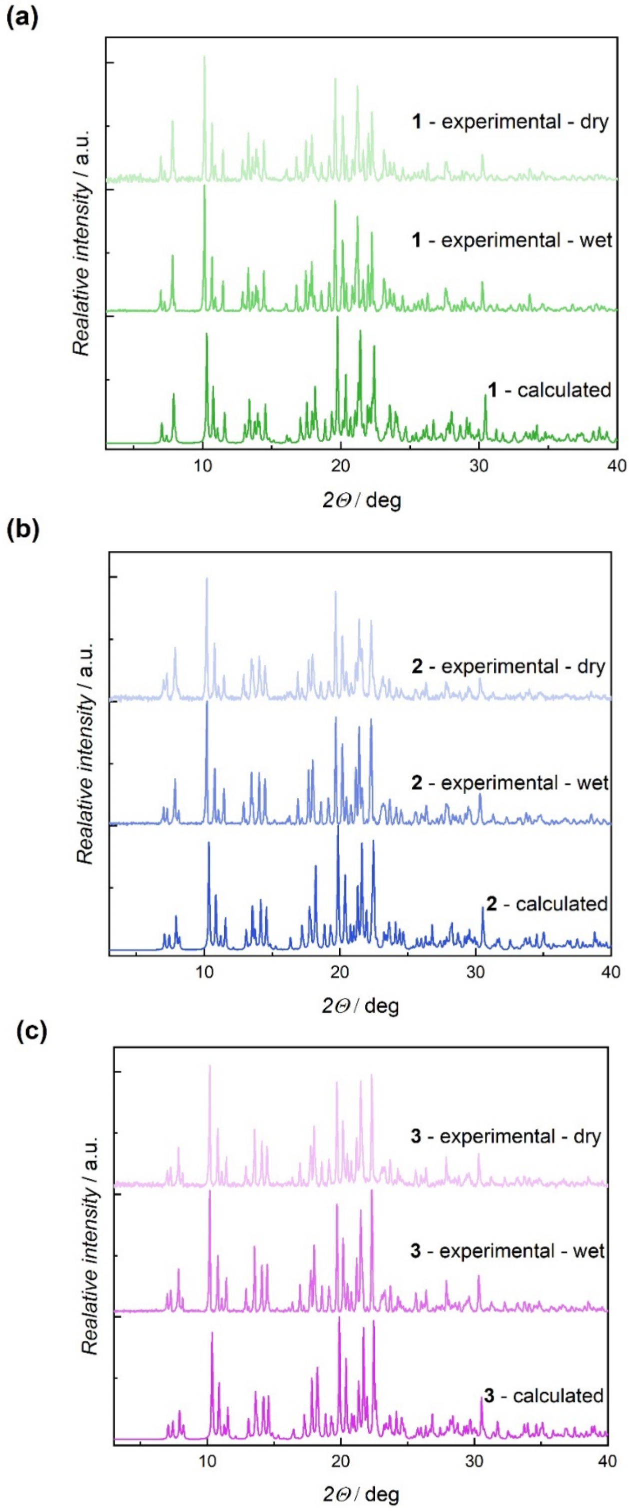

Figure S1. Powder X-ray diffraction (PXRD) patterns for **1** (a), **2** (b), **3** (c) in 3-40° range of 2Θ angle. Calculated patterns were obtained based on the respective structural models obtained by a single-crystal X-ray, 100K diffraction method. Experimental for sample immersed in mother solution (wet) and dried samples (dry), RT.

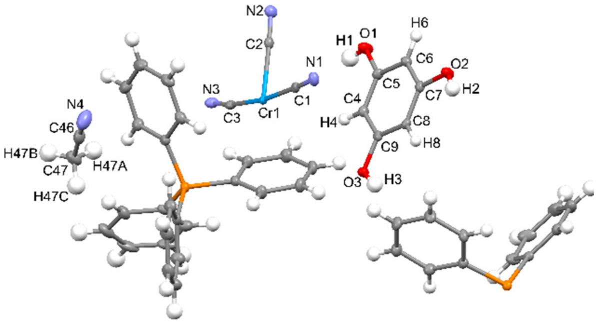

1

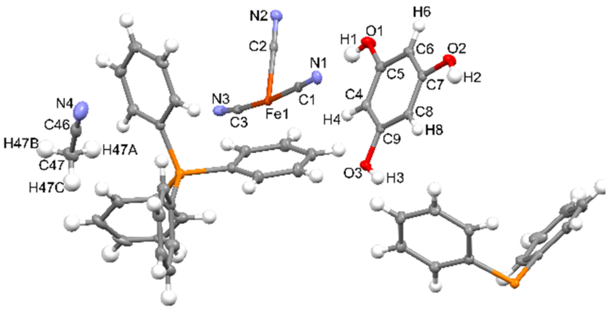

2

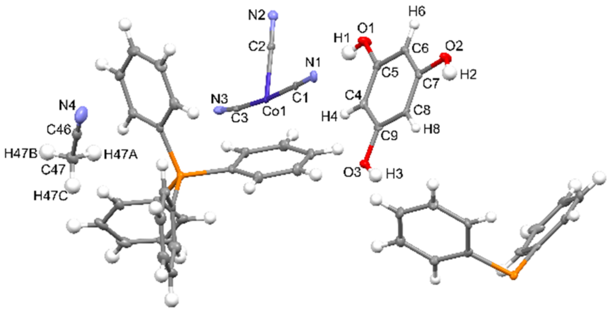

3

Figure S2. Asymmetric units of **1**-**3**. Colors: turquoise – Cr, dark orange – Fe, dark purple – Co, grey – C, blue – N red – O, orange – P, white – H. Thermal ellipsoid are drawn at the 50 % probability level.

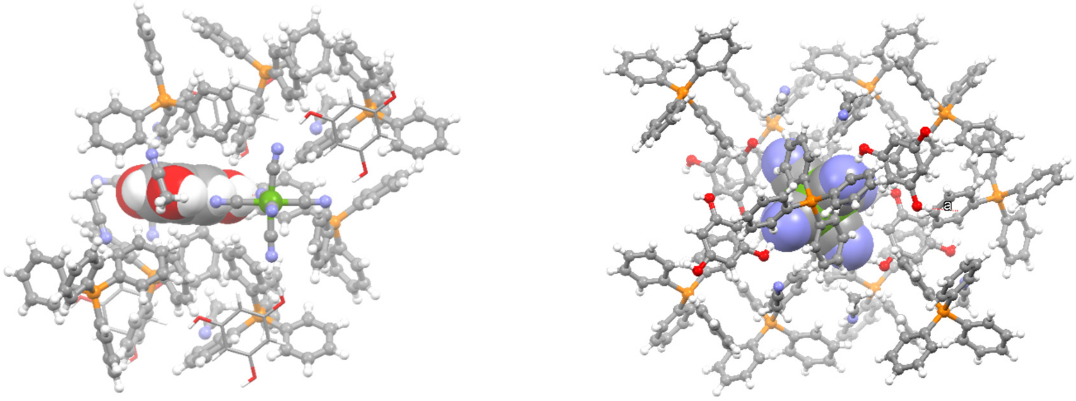

(a) (b)

Figure S3. Crystal structure of **1-3**: molecular surrounding of H_3PG (a) and of $[M(CN)_6]^{3-}$ (b). Legend: green – Cr, Fe or Co, grey – C, blue – N red – O, orange – P, white – H.

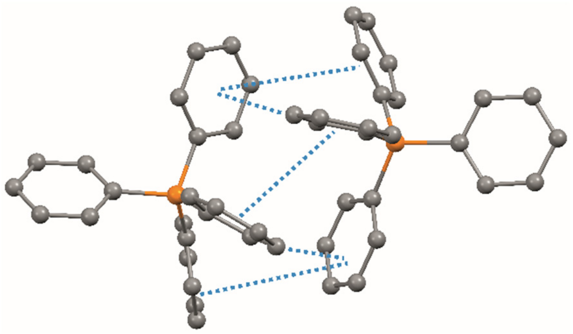

OSPE ca. 6.2 Å

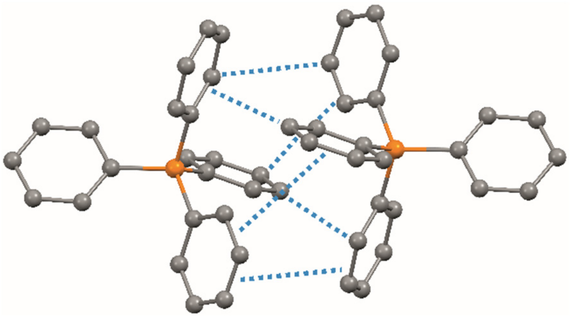

SPE ca. 6.6 Å

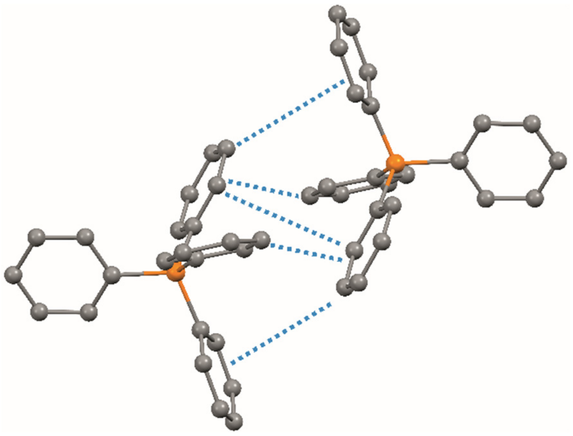

SPE ca. 7.3 Å

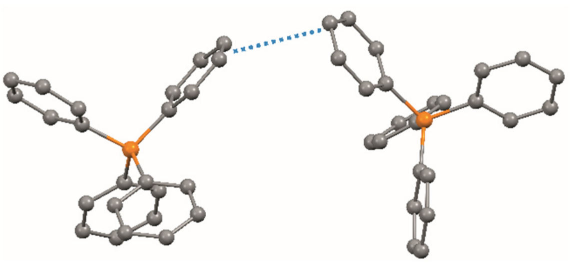

DPE ca. 9.5 Å

Figure S4. The shortest Multiple Phenyl Embrace (MPE) interactions in **1-3** (see **Figure S3**; OSPE – offset sextuple phenyl embraces; SPE – multiple phenyl embrace; DPE – double phenyl embraces [1]).

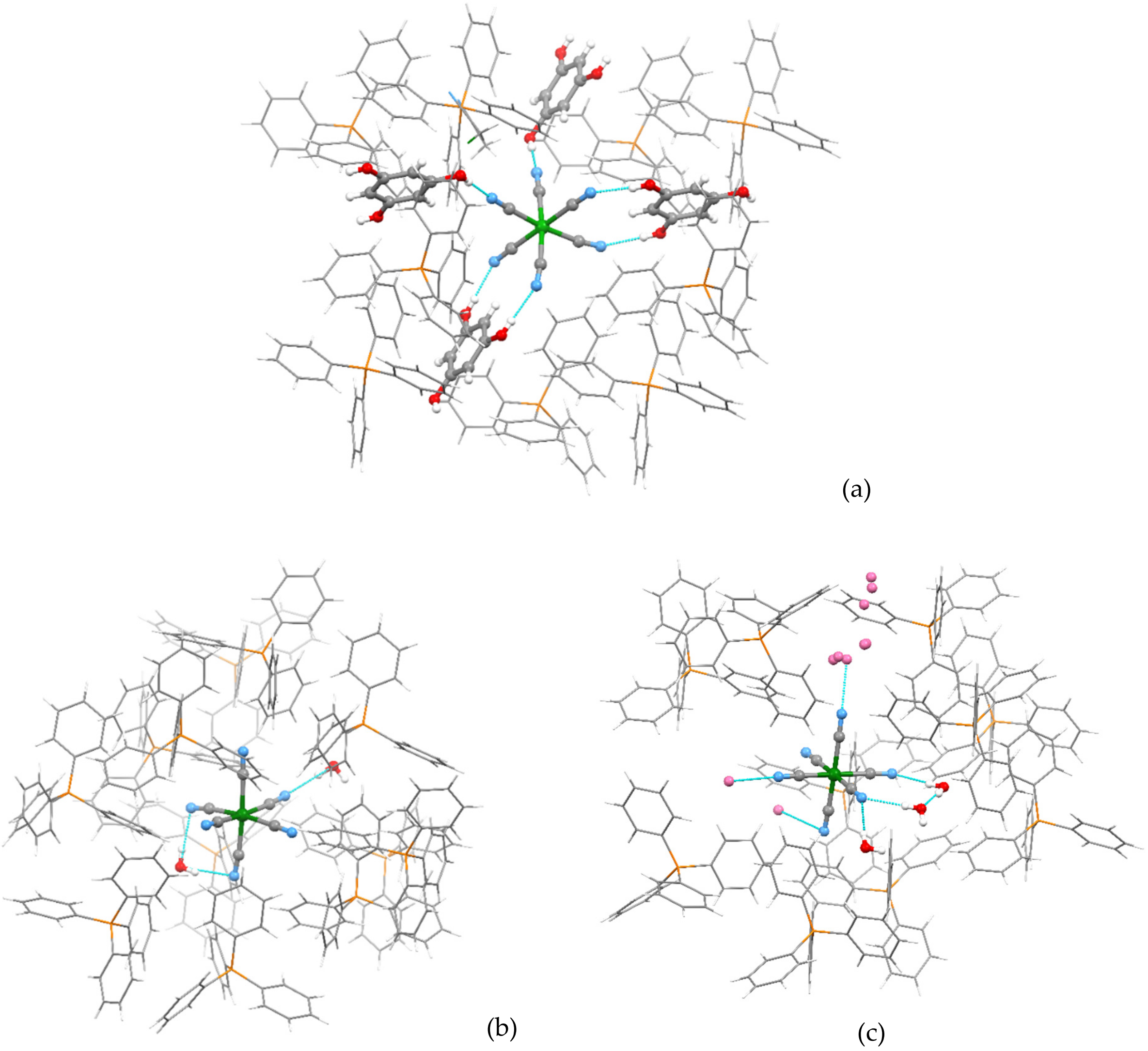

(a)

(b) (c)

Figure S5. Comparison of the molecular surrounding of $[M(CN)_6]^{3-}$ (M = Cr, Fe, Co) and hydrogen bonds in crystal structure of (a) **1**-**3** and precursors: (b) $(PPh_4)_3[Cr(CN)_6]\cdot 2H_2O$ (refcode SEGFAM) [2] and (c) $(PPh_4)_3[Fe(CN)_6]\cdot 7H_2O$ (refcode VOLVEZ) [3]. In **1**-**3** $[M(CN)_6]^{3-}$ forms six hydrogen bonds with a relatively strong Brønsted acid (H_3PG) providing the saturation of N···H-O_{ring} contacts, which contrasts with rather unsaturated second coordination sphere of $[M(CN)_6]^{3-}$ anions in the simple hydrate salts due to the N···H-O_{water} contacts involving water molecules as a relatively weaker proton donor. The relevant N···O distances in the latter contacts are statistically shorter compared those in **1**-**3**: 2.99 Å, 3.00 Å and 3.05 Å in $(PPh_4)_3[Cr(CN)_6]\cdot 2H_2O$ (b), and 2.71 Å, 2.81 Å, 2.91 Å and 2.99 Å (twice) and 3.01 Å in $(PPh_4)_3[Fe(CN)_6]\cdot 7H_2O$ (c). Legend: green – Cr, Fe or Co, grey – C, blue – N red – O, pink – disordered O of the crystallization H_2O molecules (c), orange – P, white – H. The above differences are responsible for the observed notable modifications of the vibrational, Mössbauer, electronic and photoluminescent spectral characteristics.

Table S1. Crystal data and structure refinement of **1-3**.

**Compound**	**1**	**2**	**3**
Formula	$C_{94}H_{78}CrN_8O_6P_3$	$C_{94}H_{78}FeN_8O_6P_3$	$C_{94}H_{78}CoN_8O_6P_3$
Formula weight [g·mol^{-1}]	1560.55	1564.40	1567.48
Temperature [K]	100.0	100.0	100.0
λ/Å	0.71073 Å (MoK$_\alpha$)	0.71073 Å (MoKα)	0.71073 Å (MoKα)
Crystal system	monoclinic	monoclinic	monoclinic
Space group	C2/c	C2/c	C2/c
Unit cell			
a [Å]	22.0120(5)	21.6717(7)	21.5337(5)
b [Å]	15.2472(4)	15.2992(6)	15.3238(3)
c [Å]	23.9086(6)	23.8417(8)	23.8155(5)
β [deg]	91.651(1)	91.854(1)	92.001(1)
Volume [Å^3]	8020.9(3)	7900.8(5)	7853.8(3)
Z	4	4	4
ϱ_{calc} [g/cm^3]	1.292	1.315	1.326
Absorption coefficient [mm^{-1}]	0.264	0.314	0.342
F(000)	3260.0	3268.0	3272.0
Crystal size [mm^3]	0.48×0.42×0.26	0.61×0.41×0.25	0.13×0.12×0.09
2Θ range [deg]	5.344 to 52.044	5.326 to 52.042	4.680 to 50.054
hkl ranges	-27 ≤ h ≤ 27, -18 ≤ k ≤ 18, -29 ≤ l ≤ 29	-26 ≤ h ≤ 26, -18 ≤ k ≤ 18, -29 ≤ l ≤ 29	-25 ≤ h ≤ 25, -18 ≤ k ≤ 18, -28 ≤ l ≤ 28
Reflections collected	68704	48079	46653
Independent reflections	7902 [Rsigma = 0.0157]	7779 [R_{sigma} = 0.0203]	6940 [R_{sigma} = 0.0467]
R_{int}	0.0278	0.0293	0.0696
Completeness / %	99.8	99.9	99.9
Data/restraints/parameters	7902/0/519	7779/0/510	6940/6/519
GOOF on F^2	1.107	1.090	1.147
Final R indexes [I>=2σ (I)]	R1 = 0.0350, wR2 = 0.0822	R_1 = 0.0354, wR_2 = 0.0794	R_1 = 0.0589, wR_2 = 0.1147
Final R indexes [all data]	R1 = 0.0377, wR2 = 0.0836	R_1 = 0.0399, wR_2 = 0.0812	R_1 = 0.0736, wR_2 = 0.1200
Largest diff. peak/hole [e · Å^{-3}]	0.34/-0.55	0.42/-0.45	0.56/-0.58

Table S2. The most important bond lengths and angles in **1**-**3**.

Compound	**1**	**2**	**3**
	Bond lengths [Å] and angles [deg.]		
M-C[a]	2.068	1.941	1.898
	2.070	1.940	1.902
	2.080	1.948	1.904
C≡N[a]	1.151	1.152	1.147
	1.152	1.153	1.142
	1.153	1.155	1.150
M-C≡N[a]	176.72	177.35	177.63
	176.70	177.62	177.89
	171.31	173.53	173.51
"right" C-M-C angles[b]	91.37	91.02	91.72
	88.56	92.31	93.49
	90.18	91.13	85.87
	88.39	89.24	88.81
	95.37	94.55	89.27
	93.49	88.55	91.08
	84.87	85.19	91.38
"straight" C-M-C angles	173.11 (2x)	174.42 (2x)	175.13 (2x)
	179.65	179.63	179.08

[a]The order of values is maintained following the numbering in the crystallographic file.
[b]The table lists 7 angle values, 5 of which are repeated.

Table S3. The CShM analysis of the octahedral compounds **1**-**3**.

Compound	Shape measure[a]				
	**HP**	**PPY**	**OC**	**TPR**	**JPPY**
**1**	32.696	27.507	0.287	14.788	31.056
**2**	32.612	28.006	0.208	14.904	31.587
**3**	32.865	28.261	0.150	15.258	31.962

[a]Shape symbols: HP – hexagon, PPY – pentagonal pyramid, OC – octahedron, TPR – trigonal prism, JPPY – Johnson pentagonal pyramid.

2. Hirshfeld analysis

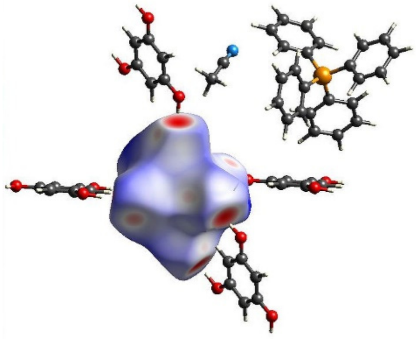

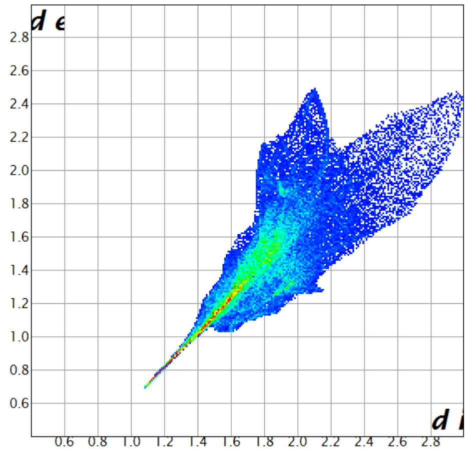

(a) (b)

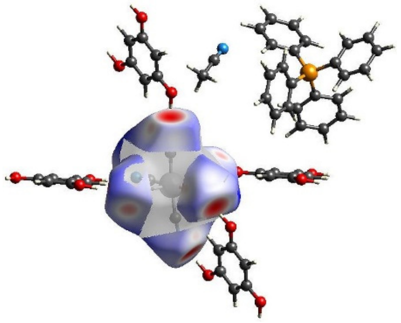

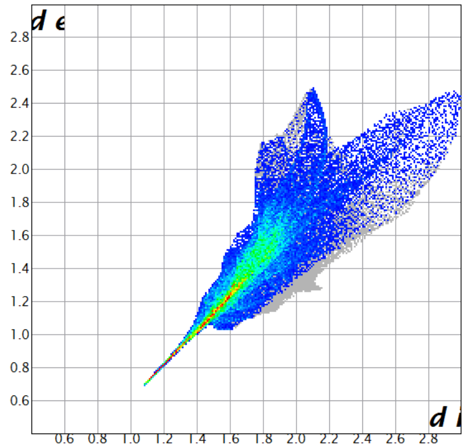

(c) (d)

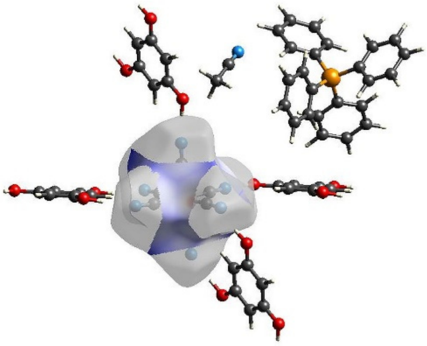

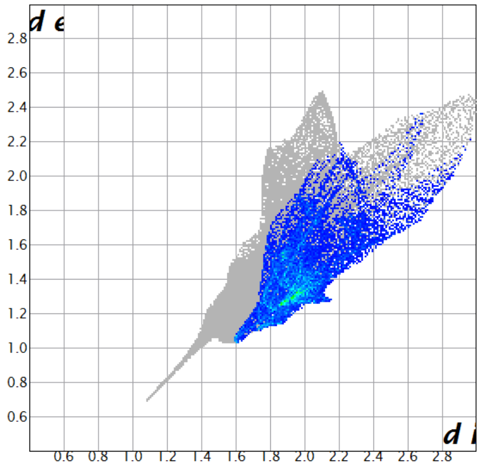

(e) (f)

Figure S6. Hirshfeld surface (left column) for $[Fe(CN)_6]^{3-}$ with projected selected interactions and corresponding fingerprints (right column): (a) and (b) – all interactions; (c) and (d) – N···H interactions (74.4%); (e) and (f) – C···H (21.4%).

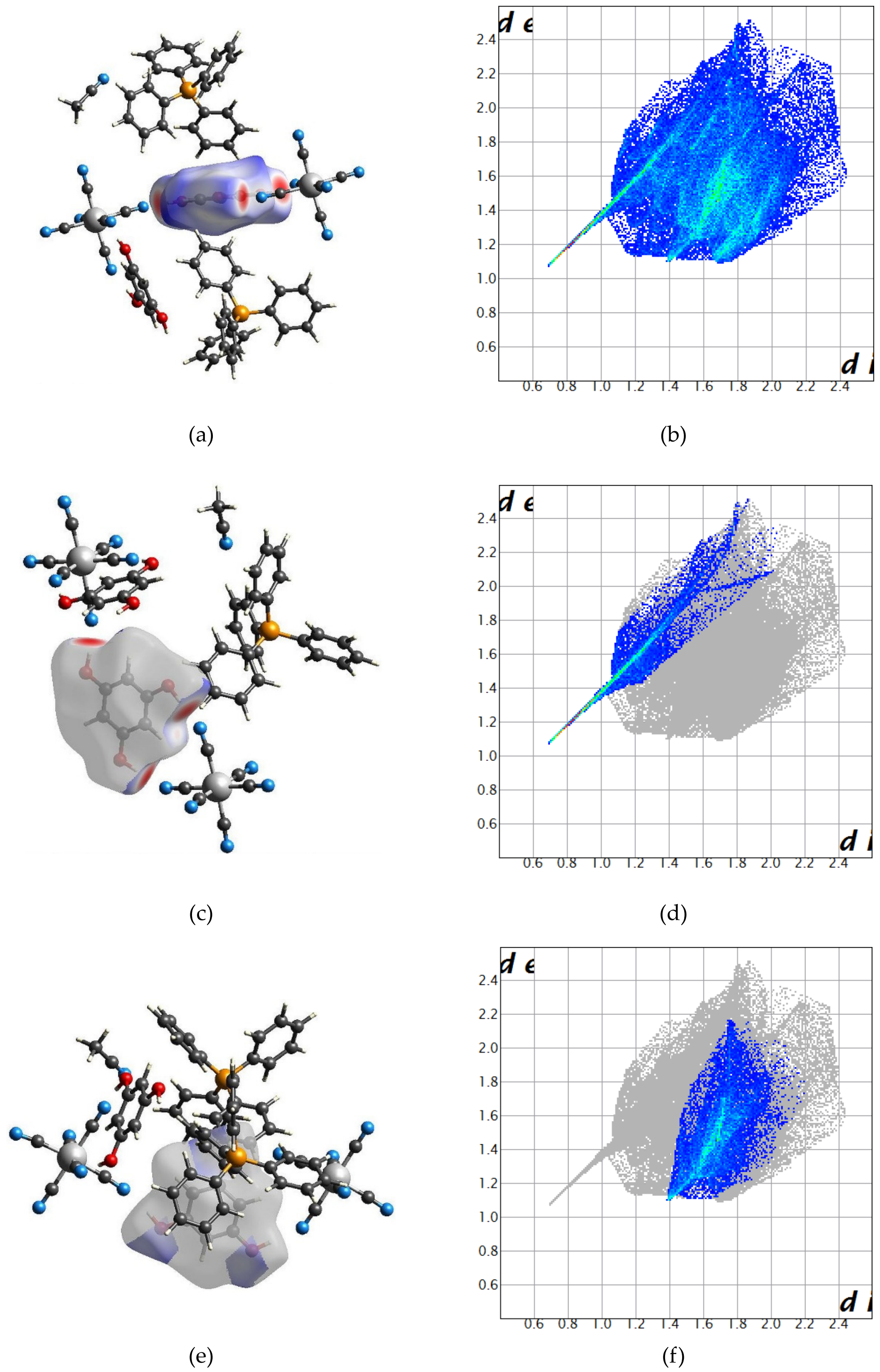

Figure S7. Hirshfeld surface (left column) for H_3PG with projected selected interactions and corresponding fingerprints (right column): (a) and (b) – all interactions, (c) and (d) – H···N interactions (14.7%); (e) and (f) – O···H (23.1%).

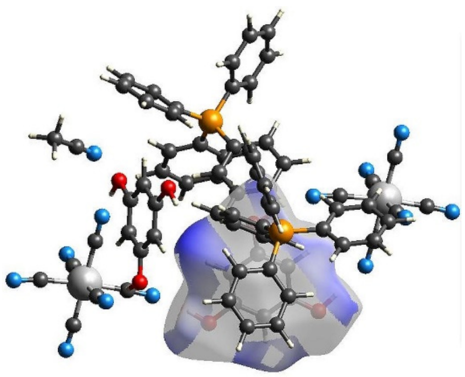

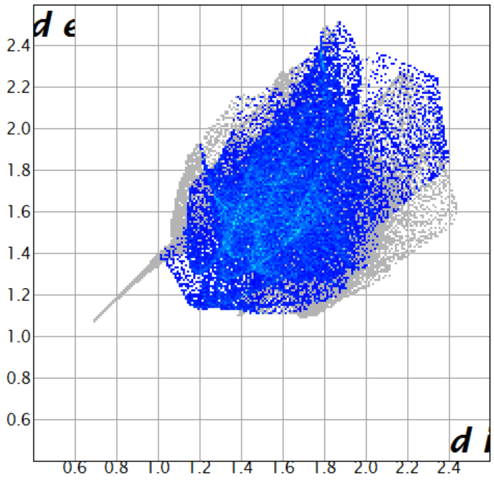

(g) (h)

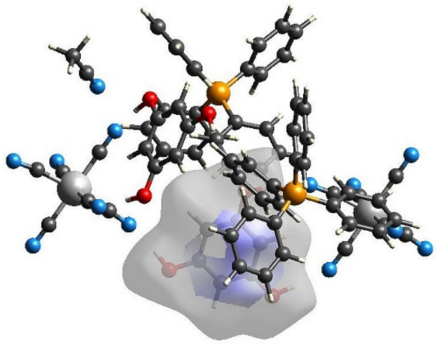

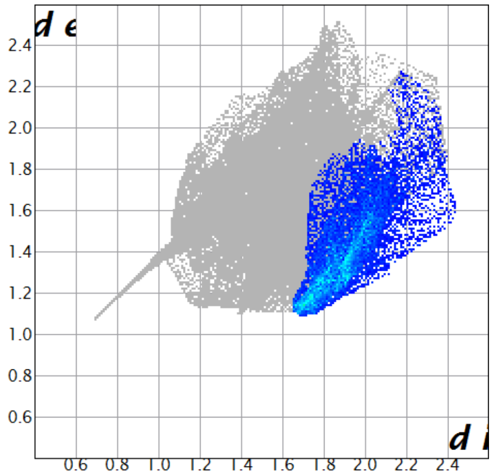

(i) (j)

Figure S7 continued: (g) and (h) – H···H (38.2%); (i) and (j) – C···H (18.3%).

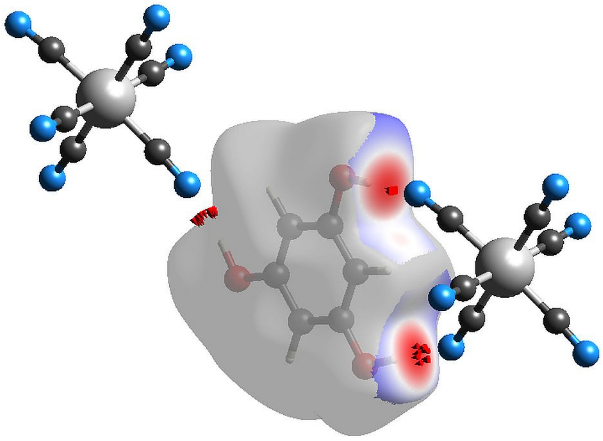

(a)

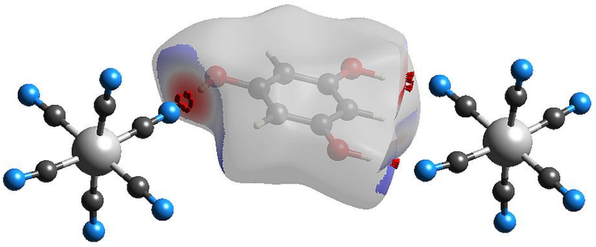

(b)

Figure S8. Hirshfeld surface for H_3PG and $[Fe(CN)_6]^{3-}$ with projected selected interactions: double (a) and single (b) synthons. Red arrows correspond to interactions related to the spike on the fingerprint in **Figure S6**.

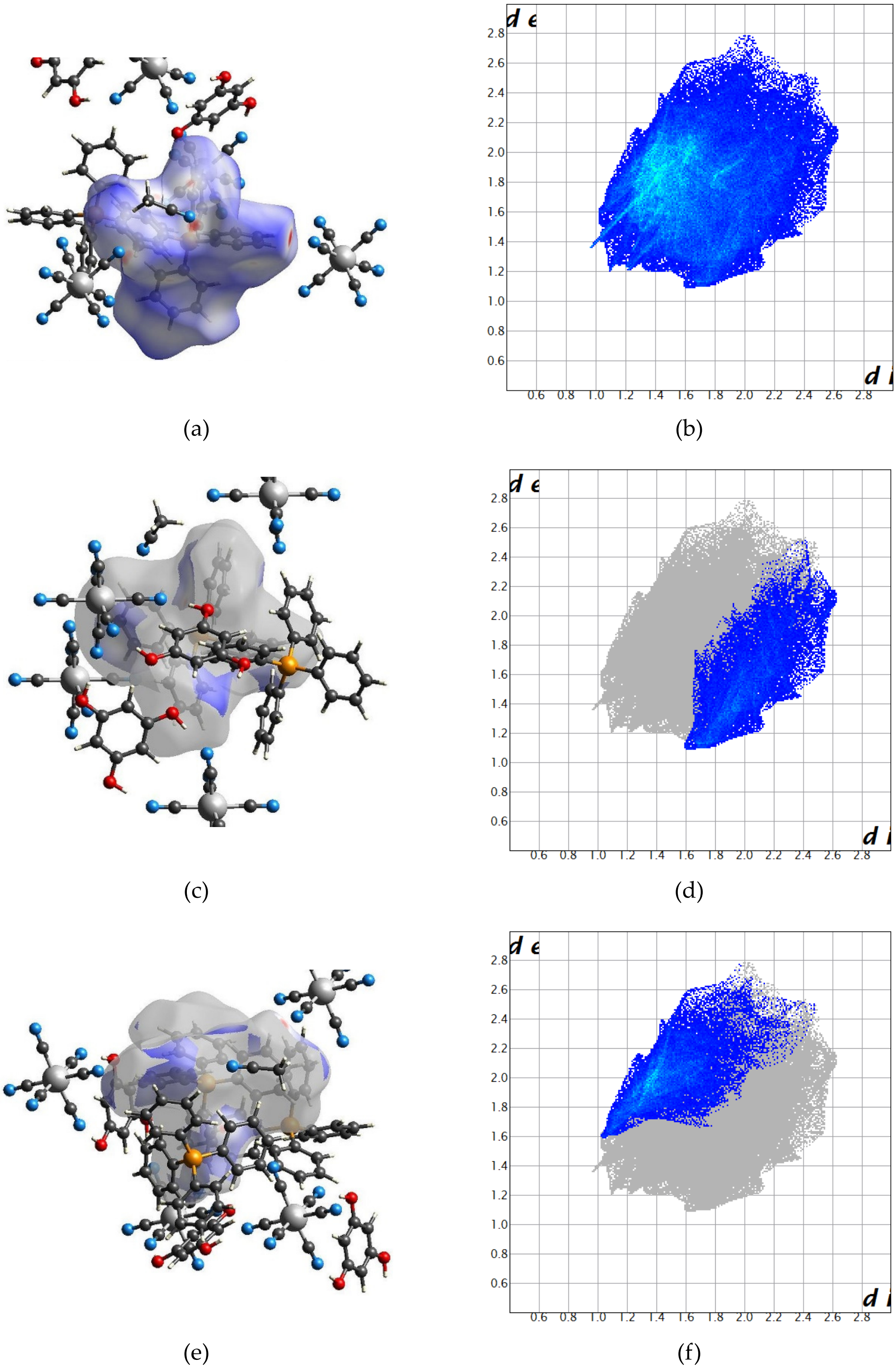

Figure S9. Hirshfeld surface (left column) for $P(1)Ph_4^+$ with projected selected interactions and corresponding fingerprints (right column): (a) and (b) – all interactions; (c) and (d) C···H interactions (15.9%, first letter – in this case C – denotes atom type inside the surface, and the second – here H – atom type outside the surface); (e) and (f) – H···C (19.3%).

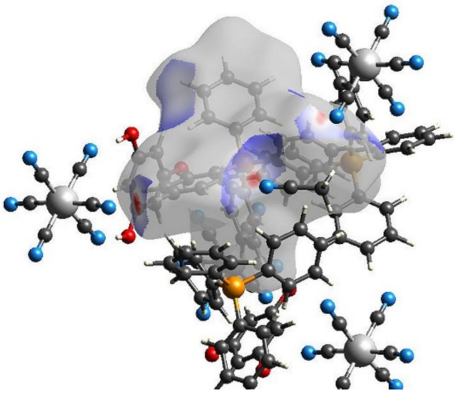

(g)

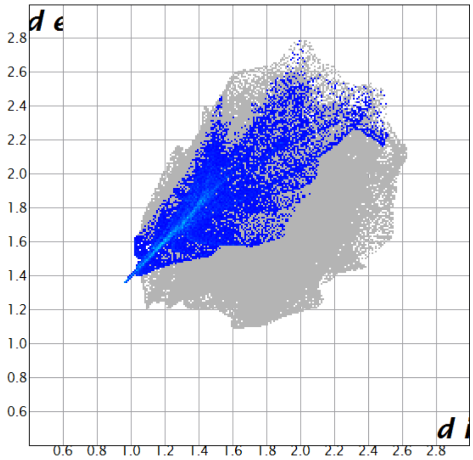

(h)

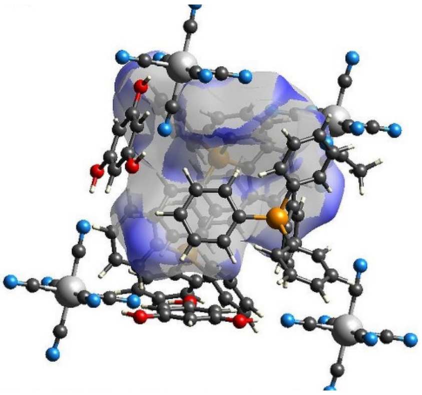

(i)

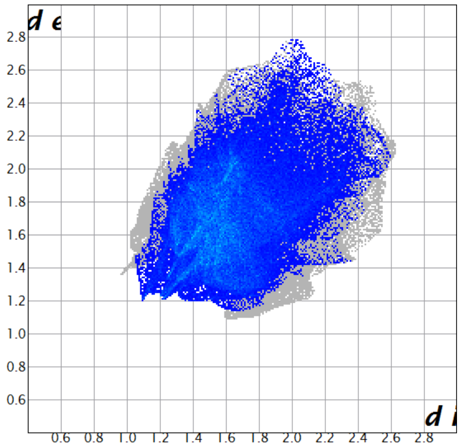

(j)

Figure S9 continued: (g) and (h) – H···N (10.3%); (i) and (j) – H···H (45.7%).

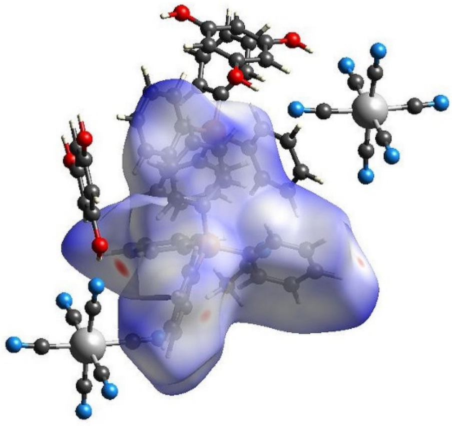

(a)

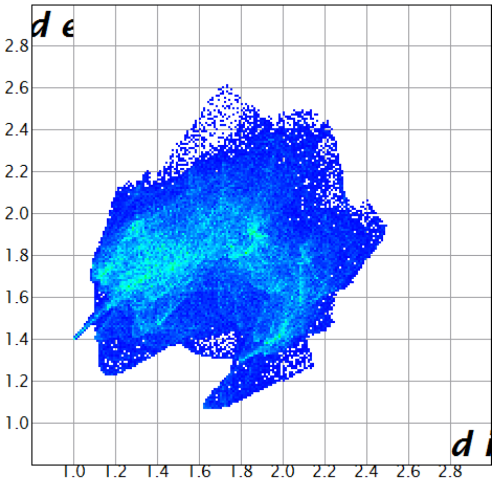

(b)

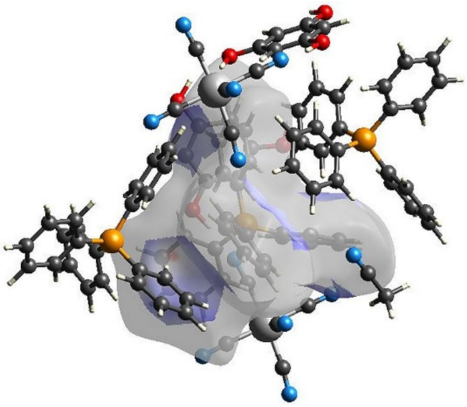

(c)

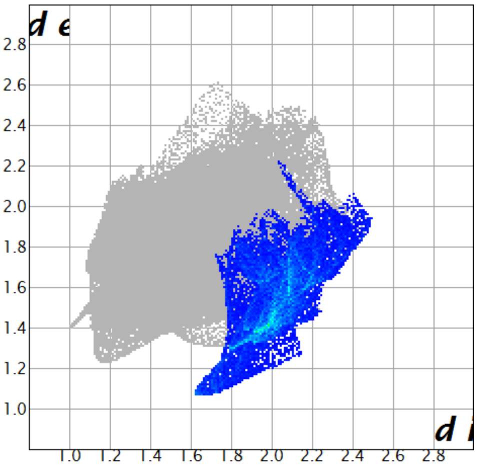

(d)

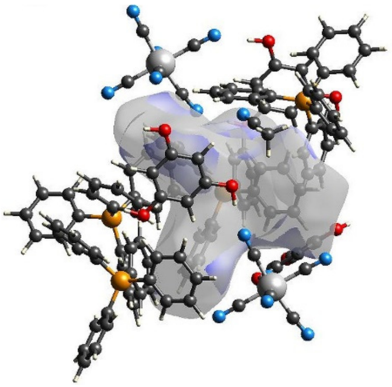

(e)

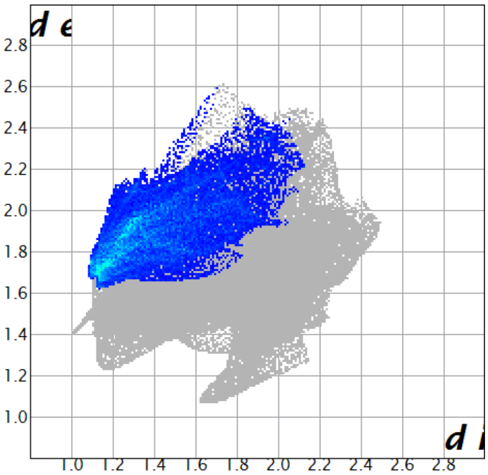

(f)

Figure S10. Hirshfeld surface (left column) for $(P(2)Ph_4)^+$ with projected selected interactions and corresponding fingerprints (right column): (a) and (b) – all interactions; (c) and (d) – C···H interactions (16.7%); (e) and (f) – H···C (21.9%).

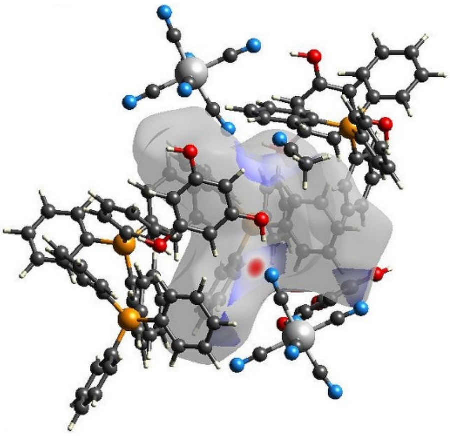

(g)

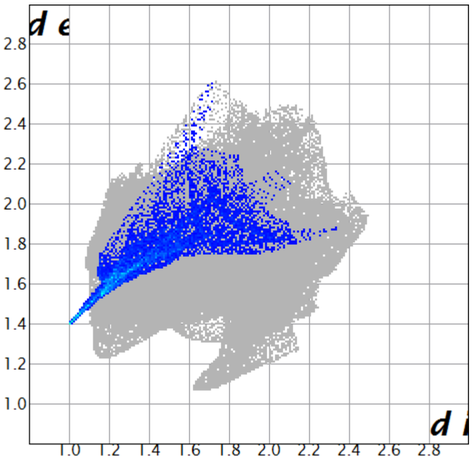

(h)

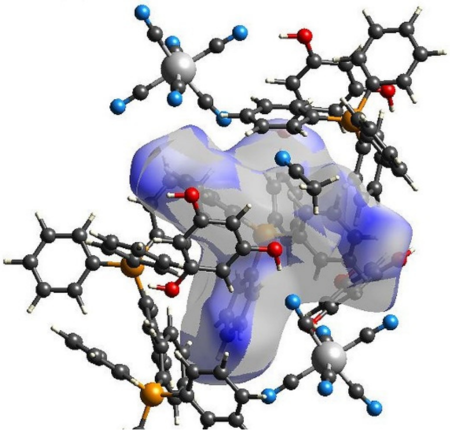

(i)

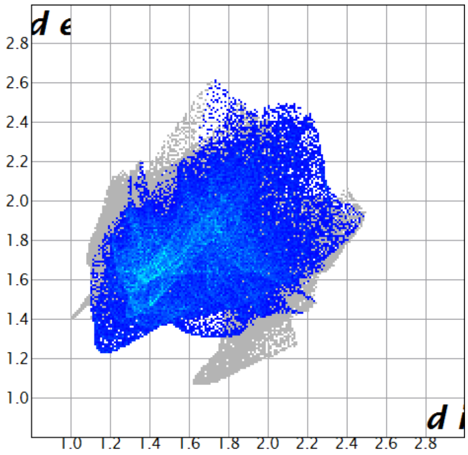

(j)

Figure S10 continued: (g) and (h) – H···N (7.7%); (i) and (j) H···H (44.7%).

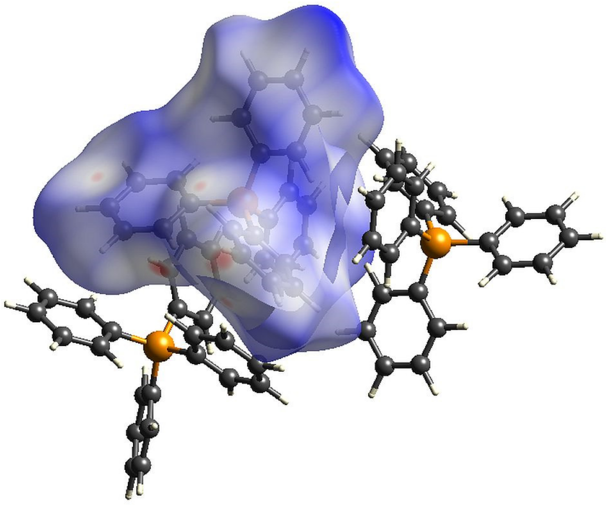

(a)

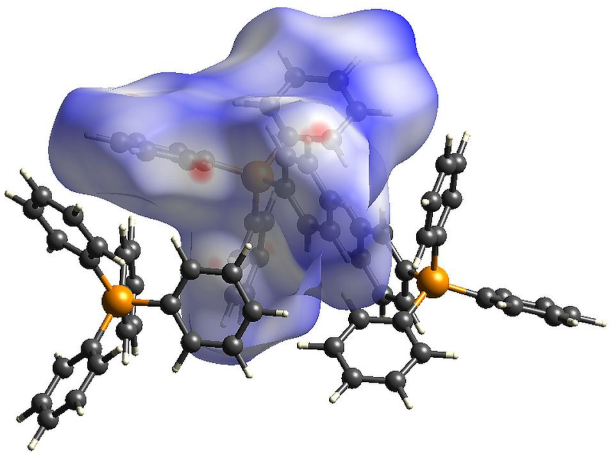

(b)

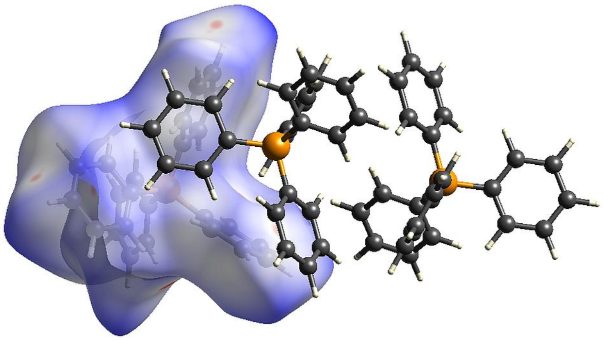

(c)

Figure S11. Hirshfeld surface presenting contacts in the PPh_4^+ columns: P1-P1 (a) P1-P2 (b) and P2-P1 (c) interface.

3. ESI-MS

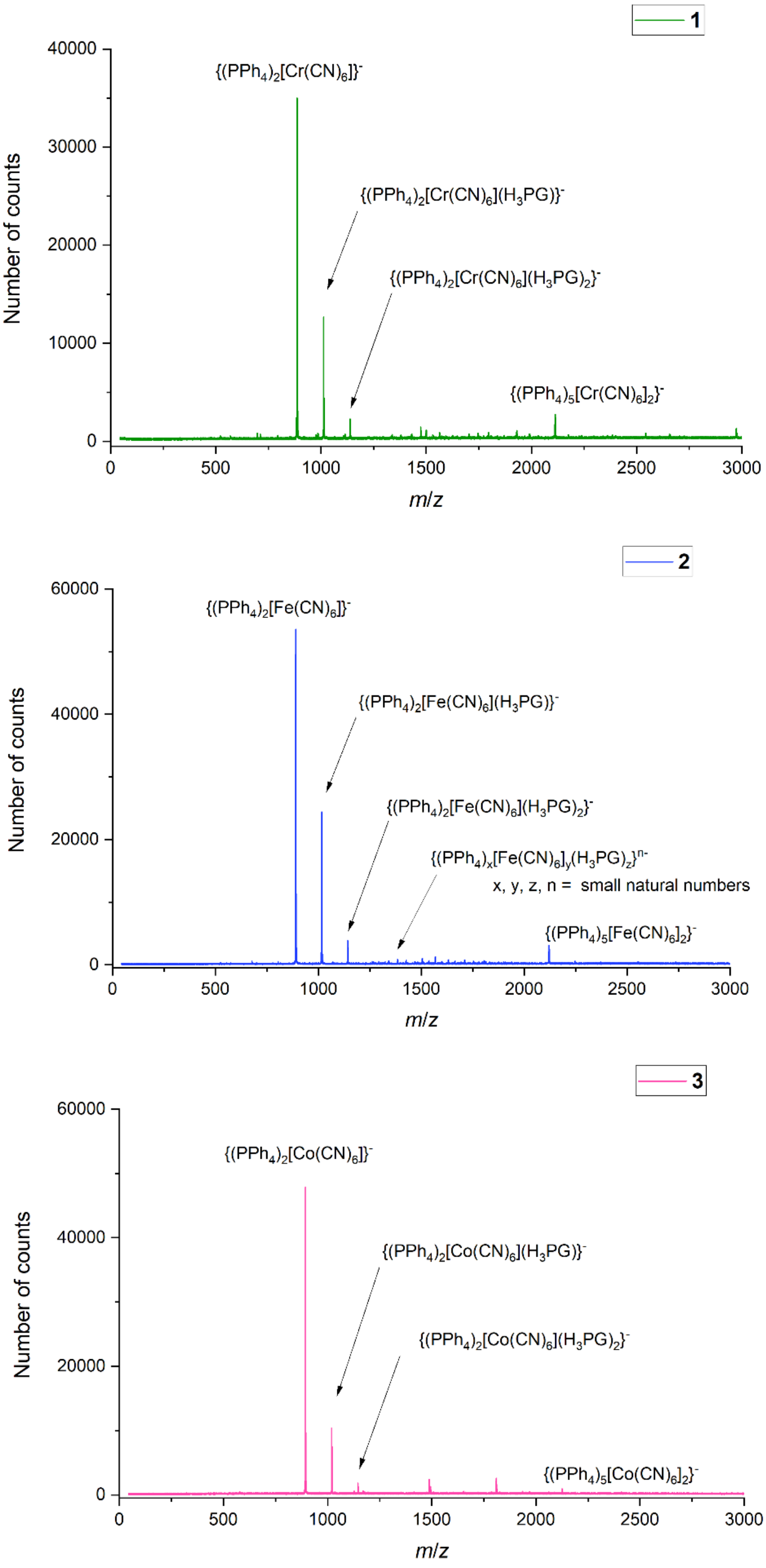

Figure S12. The whole ESI-MS spectrograms in the negative ionization mode for **1**, **2** and **3**.

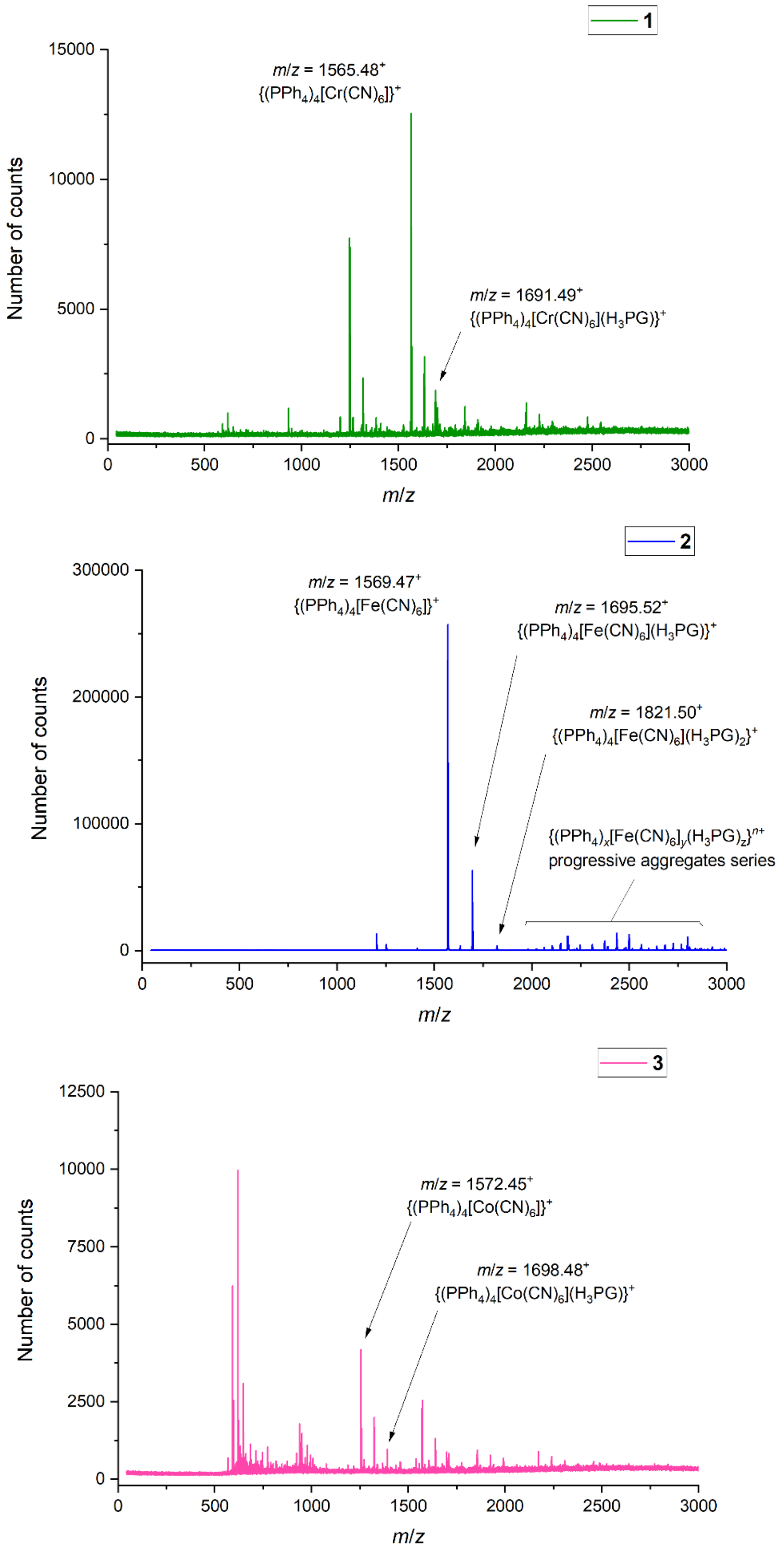

Figure S13. The whole ESI-MS spectrograms in the positive ionization mode for **1**, **2** and **3**.

4. Computational details

Calculations were performed for hydrogen-bonded molecular clusters comprising the hexacyanidometallate anion $[M(CN)_6]^{3-}$ (M = Cr, Fe, Co) and four or one phloroglucinol molecule(s) PGH_3, $\{[M(CN)_6]^{3-};(\mathbf{H}_2PGH)_2(\mathbf{H}PGH_2)_2\}$, $\{[M(CN)_6]^{3-};\mathbf{H}_2PGH\}$, and $\{[M(CN)_6]^{3-};\mathbf{H}PGH_2\}$, extracted from the crystal structures of **1**, **2**, and **3**, and, for a comparison, for hydrogen-bonded molecular clusters comprising *trans*-1,2-bis(4-pyridyl)ethylene (dpe) and PGH_3 ($\{dpe;\mathbf{H}PGH_2\}$) and 4,4′-bipyridyl (4,4′bpy) and PGH_3 ($\{4,4'bpy;\mathbf{H}PGH_2\}$) extracted from the crystal structures reported in respectively Ref. [4] and Ref. [5]. For molecular clusters visualizations, see Figure S14. All the computations were carried out at the density functional theory (DFT) level using the Amsterdam Density Functional (ADF) program, version 2019.304 [6,7].
The interaction energy between the hydrogen-bond acceptor ($[M(CN)_6]^{3-}$ with a spin-quartet d^3 electronic configuration of Cr, spin-doublet d^5 of Fe, and closed-shell d^6 of Co; dpe; 4,4′bpy) and the hydrogen-bond donor PGH_3 molecule(s) in the aforementioned clusters was calculated as the difference between electronic ground-state energy of the given molecular cluster and the sum of electronic ground-state energies of its fragments (hydrogen-bond acceptor and PGH_3 molecule(s)) in the geometry of the cluster. The energy calculations were performed using dispersion-corrected DFT+D4 [8] employing exchange-correlation density functionals belonging to different classes of approximation, BLYP [9,10] (gradient), B3LYP [10-12] (global hybrid), rev-DOD-BLYP and rev-DSD-BLYP [13] (double hybrids), a relativistic all-electron triple-ξ singly (TZP) and doubly polarized (TZ2P) basis sets from the ADF, and without and with continuum solvent model (conductor like screening model = COSMO [14,15]) to simulate acetonitrile effects. Scalar relativistic effects were modelled by using the zeroth-order regular approximation (ZORA) [16,17].
ETS-NOCV [18,19] charge and energy decomposition analyses (vide infra) were carried out on (closed-shell) $\{[Co(CN)_6]^{3-};\mathbf{H}_2PGH\}$ and $\{[Co(CN)_6]^{3-};\mathbf{H}PGH_2\}$, and reference $\{4,4'bpy;\mathbf{H}PGH_2\}$ and $\{dpe;\mathbf{H}PGH_2\}$ molecular clusters. These calculations were performed in gas-phase with BLYP+D4//TZP using ZORA.
The interaction energy, ΔE_{int}, between the fragments in the geometry of the system can be divided using the extended transition state (ETS) approach into following components:

$$\Delta E_{int} = \Delta E_{orb} + \Delta E_{Pauli} + \Delta E_{elstat} \left(+\Delta E_{disp}\right)$$

where:

ΔE_{orb} – corresponds to the stabilizing interactions between the occupied molecular orbitals on one fragment with the unoccupied molecular orbitals of the other fragment (describing inter-fragments donation and back-donation charge transfers), as well as the mixing of occupied and virtual orbitals within the same fragment (describing intra-fragment polarization, charge redistribution),

ΔE_{Pauli} – accounts for the repulsive Pauli interaction between occupied orbitals on the two fragments,

ΔE_{elstat} – represents the classical electrostatic interaction between the fragments in the combined system,

ΔE_{disp} – corresponds to the dispersion interaction between the fragments in the combined system, and herein was obtained at the DFT+D4 level.

The natural orbitals for chemical valence (NOCV) are eigenvectors that diagonalize the deformation density matrix in the basis of fragment orbitals. The NOCV pairs (ψ_{-k}, ψ_k with eigenvalues respectively

$-v_k$ and v_k, that is of the same absolute value but opposite signs) decompose the deformation density $\Delta\rho$ into NOCV contributions, $\Delta\rho_k$:

$$\Delta\rho = \sum_k \Delta\rho_k = \sum_k v_k(-\psi_{-k}^2 + \psi_k^2)$$

with k going over the pairs of NOCV's. $\Delta\rho_k$ plots can be analyzed visually to assign symmetry and the direction of the density flow.

Finally, within the ETS-NOCV scheme, the orbital interaction term ΔE_{orb} is further expressed in the NOCV representation as a sum of the orbital energy contributions ΔE_k corresponding to the particular $\Delta\rho_k$ channels.

Additional calculated data

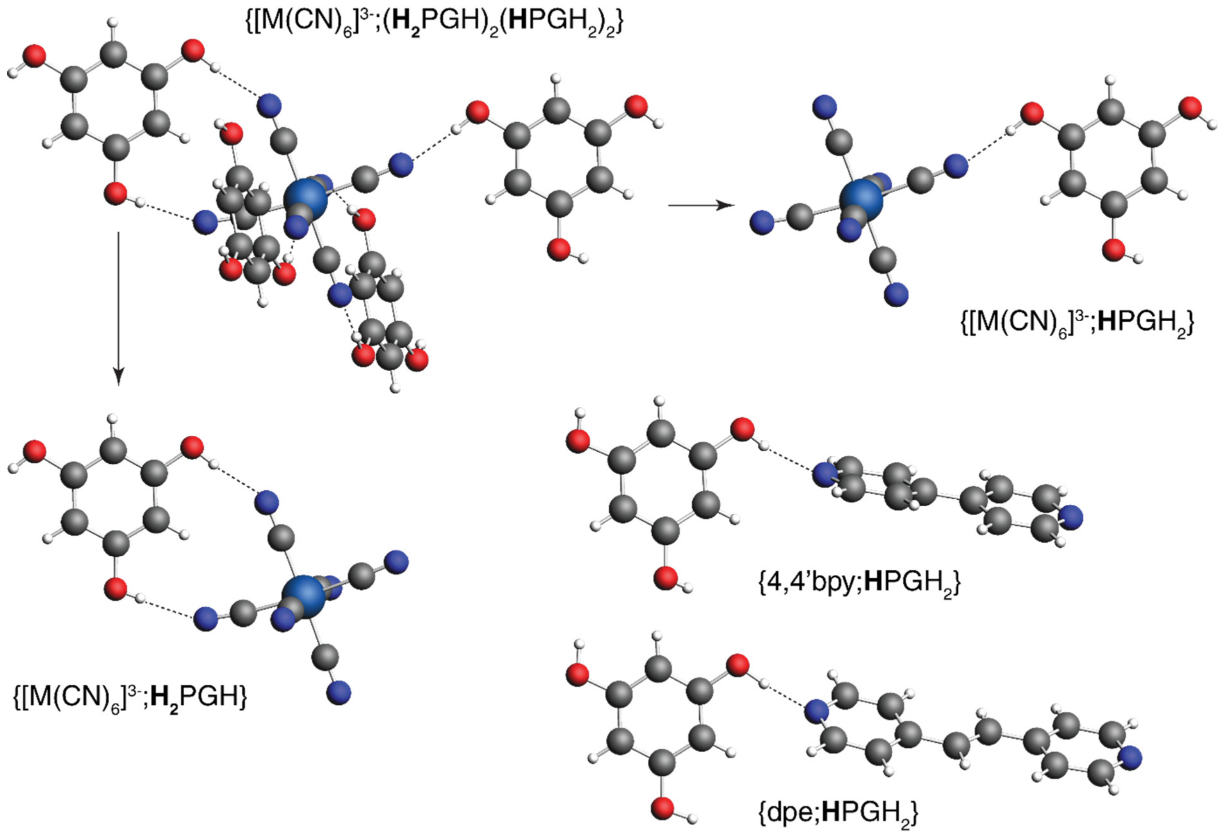

Figure S14. Visualization of the examined molecular clusters $\{[M(CN)_6]^{3-};(\mathbf{H}_2PGH)_2(\mathbf{H}PGH_2)_2\}$, $\{[M(CN)_6]^{3-};\mathbf{H}_2PGH\}$, and $\{[M(CN)_6]^{3-};\mathbf{H}PGH_2\}$ with M = Cr, Fe, Co (shown), $\{dpe;\mathbf{H}PGH_2\}$ and $\{4,4'bpy;\mathbf{H}PGH_2\}$.

Table S4. DFT-computed interaction energy values (in kcal/mol) between $[M(CN)_6]^{3-}$ (M = Cr, Fe, Co) anion and H_3PG molecule(s) in molecular clusters {$[M(CN)_6]^{3-}$;(**H**$_2$PGH)$_2$(**H**PGH$_2$)$_2$}, {$[M(CN)_6]^{3-}$;**H**$_2$PGH}, and {$[M(CN)_6]^{3-}$;**H**PGH$_2$} extracted from the crystal structures of **1**, **2**, and **3**. For a comparison, the corresponding interaction energies between *trans*-1,2-bis(4-pyridyl)ethylene (dpe) and H_3PG in molecular cluster {dpe;**H**PGH$_2$} extracted from the crystal structure reported in Ref. [4] and between 4,4′-bipyridyl (4,4′bpy) and PGH_3 in molecular cluster {4,4′bpy;**H**PGH$_2$} extracted from the crystal structure reported in Ref. [5] are listed. For molecular clusters visualizations, see **Figure S14**. Calculations were performed using dispersion-corrected DFT+D4 employing exchange-correlation density functionals belonging to different classes of approximation, BLYP (gradient), B3LYP (global hybrid), rev-DOD-BLYP and rev-DSD-BLYP (double hybrids), TZP and TZ2P basis sets, and without and with continuum solvent model to simulate acetonitrile effects (the latter results are provided in parentheses).

	$[Cr(CN)_6]^{3-}$	$[Fe(CN)_6]^{3-}$	$[Co(CN)_6]^{3-}$	dpe	4,4′bpy
BLYP+D4//TZP					
(**H**$_2$PGH)$_2$(**H**PGH$_2$)$_2$	-125.33 (-18.66)	-133.58 (-21.09)	-131.42 (-19.48)	–	–
**H**$_2$PGH	-44.98 (-7.32)	-48.44 (-8.05)	-46.74 (-7.36)	–	–
**H**PGH$_2$	† (-2.50)	-27.30 (-2.79)	-24.70 (-2.76)	-11.27 (-9.05)	-7.64 (-4.81)
BLYP+D4//TZ2P					
(**H**$_2$PGH)$_2$(**H**PGH$_2$)$_2$	-124.41 (-18.05)	-132.58 (-20.41)	-130.53 (-18.94)	–	–
**H**$_2$PGH	-44.68 (-7.14)	-48.10 (-7.84)	-46.45 (-7.23)	–	–
**H**PGH$_2$	† (-2.36)	-27.29 (-2.75)	-24.56 (-2.63)	-11.06 (-8.86)	-7.46 (-4.64)
B3LYP+D4//TZP					
(**H**$_2$PGH)$_2$(**H**PGH$_2$)$_2$	-124.87 (-18.68)	-133.38 (-21.03)	-130.88 (-19.50)	–	–
**H**$_2$PGH	-44.65 (-7.19)	-47.98 (-7.72)	-46.40 (-7.25)	–	–
**H**PGH$_2$	-22.30 (-2.42)	-24.29 (-2.71)	-23.99 (-2.69)	-11.33 (-8.88)	-7.70 (-4.74)

Table S4 continued.

B3LYP+D4//TZ2P					
($\mathbf{H}_2$PGH)$_2$($\mathbf{H}$PGH$_2$)$_2$	-124.22 (-18.24)	-132.65 (-20.61)	-130.16 (-19.13)	–	–
$\mathbf{H}_2$PGH	-44.41 (-7.09)	-47.68 (-7.53)	-46.14 (-7.15)	–	–
$\mathbf{H}$PGH$_2$	-22.17 (-2.36)	-24.16 (-2.71)	-23.87 (-2.63)	-11.17 (-8.74)	-7.56 (-4.62)
rev-DOD-BLYP+D4//TZ2P					
($\mathbf{H}_2$PGH)$_2$($\mathbf{H}$PGH$_2$)$_2$	-134.00 (-28.21)	-142.66 (-27.85)	-138.84 (-28.60)	–	–
$\mathbf{H}_2$PGH	-47.28 (-10.06)	-51.02 (-9.87)	-48.77 (-10.11)	–	–
$\mathbf{H}$PGH$_2$	-23.99 (-4.55)	-26.60 (-4.72)	-26.46 (-5.58)	-13.14 (-10.58)	-9.50 (-6.46)
rev-DSD-BLYP+D4//TZ2P					
$\mathbf{H}_2$PGH	-47.01 (-9.99)	-50.16 (-10.41)	-48.58 (-9.85)	–	–
$\mathbf{H}$PGH$_2$	-23.84 (-4.33)	-25.82 (-4.78)	-26.53 (-5.57)	-13.02 (-10.48)	-9.35 (-6.33)

† Calculations failed to reach SCF convergence for the motif.

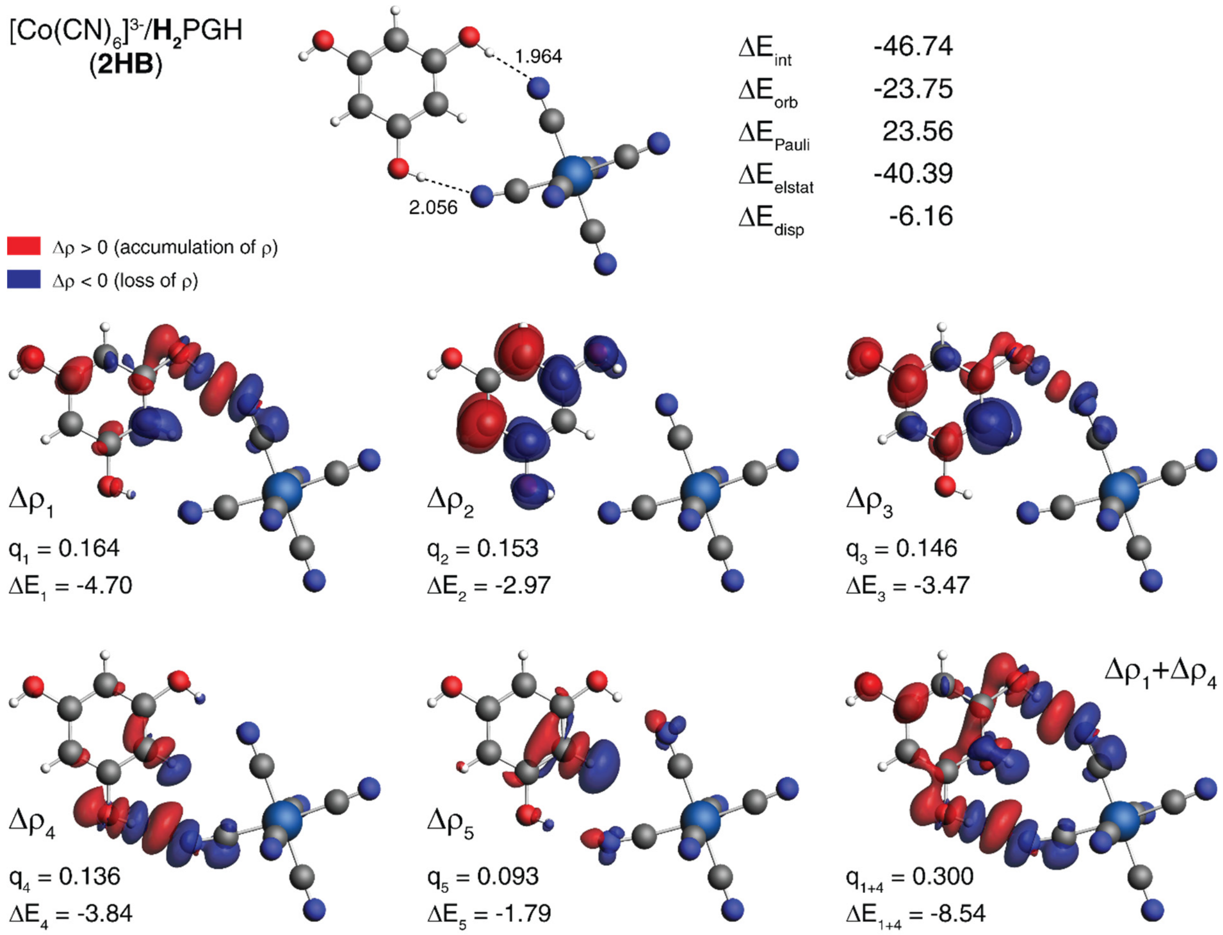

Figure S15. ETS-NOCV analysis of the interaction between $[Co(CN)_6]^{3-}$ and H_3PG in the molecular cluster $\{[Co(CN)_6]^{3-};\mathbf{H_2}PGH\}$ extracted from the crystal structure of **3**. Top: Molecular cluster $\{[Co(CN)_6]^{3-};\mathbf{H_2}PGH\}$ with the hydrogen-bond distances, in Å, listed. $[Co(CN)_6]^{3-}/H_3PGH$ energy interaction values and their components (in kcal/mol) according to the ETS energy decomposition scheme. Bottom: Isosurfaces (±0.0005 au) of five largest NOCV contributions to charge deformation (differential) density $\Delta\varrho$ along with their corresponding charge (q in e) and orbital energy (ΔE in kcal/mol) assessment. Red / blue indicates inflow (gain) / outflow (loss) of electron density. Based on BLYP+D4//TZP calculations.

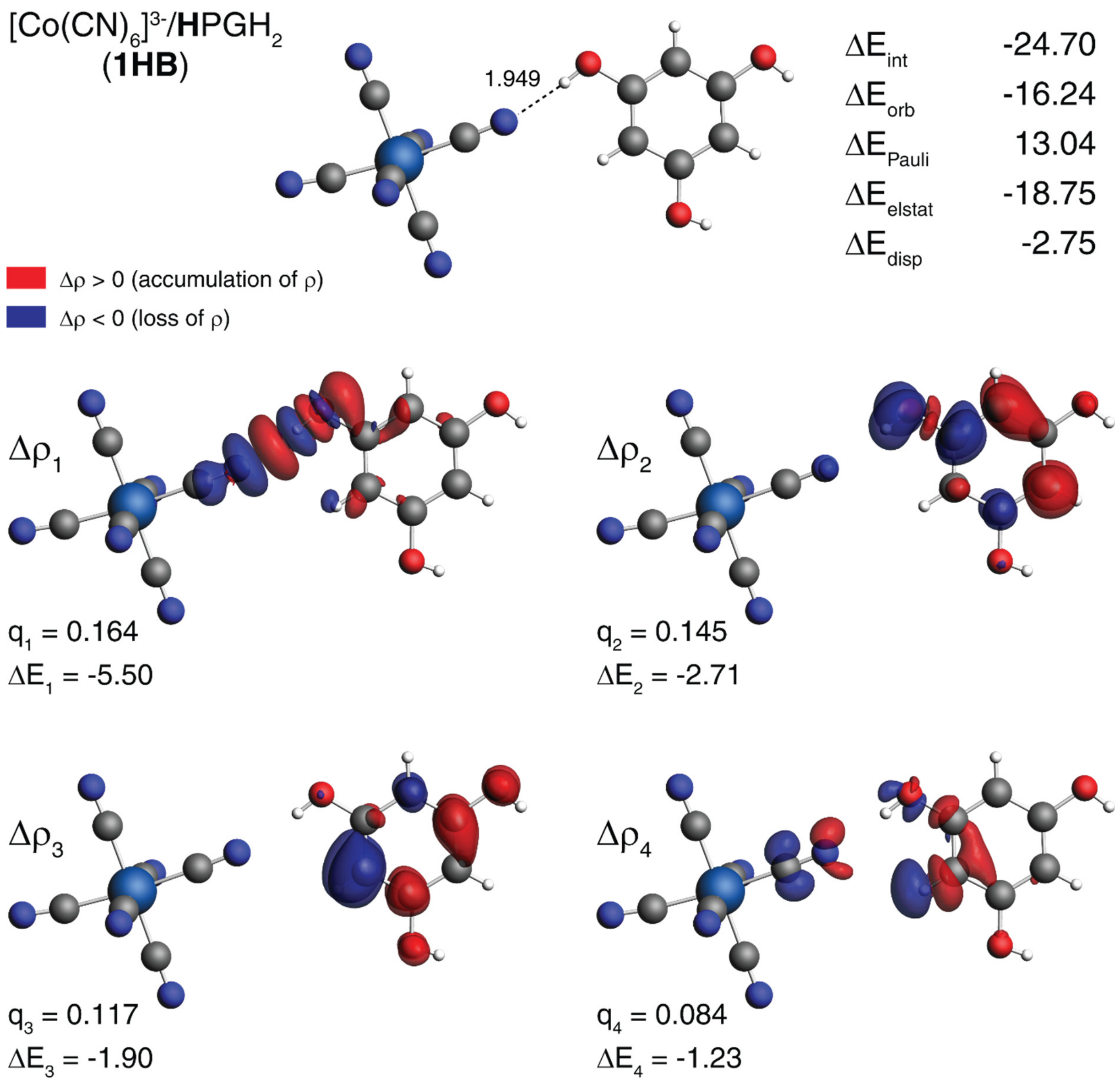

Figure S16. ETS-NOCV analysis of the interaction between $[Co(CN)_6]^{3-}$ and H_3PG molecule in the molecular cluster {$[Co(CN)_6]^{3-}$;**H**PGH_2} extracted from the crystal structure of **3**. Top: Molecular cluster {$[Co(CN)_6]^{3-}$;**H**PGH_2} with the hydrogen-bond distance, in Å, listed. $[Co(CN)_6]^{3-}$/H_3PG energy interaction values and their components (in kcal/mol) according to the ETS energy decomposition scheme. Bottom: Isosurfaces (±0.0005 au) of four largest NOCV contributions to charge deformation (differential) density $\Delta\varrho$ along with their corresponding charge (q in e) and orbital energy (ΔE in kcal/mol) assessment. Red / blue indicates inflow (gain) / outflow (loss) of electron density. Based on BLYP+D4//TZP calculations.

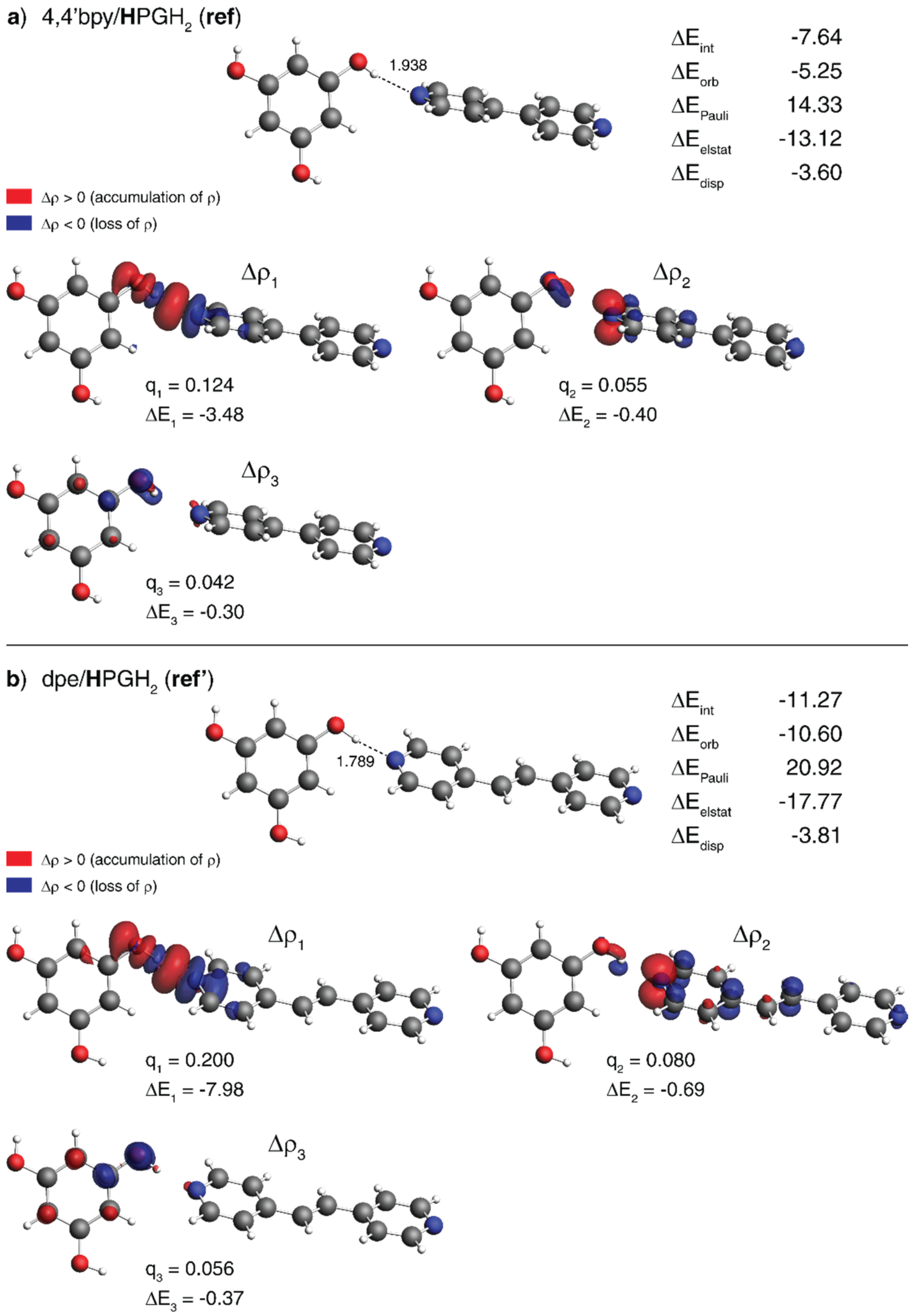

Figure S17. ETS-NOCV analysis of the interaction between 4,4′-bipyridyl (4,4′bpy) and H_3PG molecule in the molecular cluster {4,4′bpy;**H**PGH_2} extracted from the crystal structure reported in Ref. [5] (panel a) and between *trans*-1,2-bis(4-pyridyl)ethylene (dpe) and H_3PG in the molecular cluster {dpe;**H**PGH_2} extracted from the crystal structure reported in Ref. [4] (panel b). Top in each panel: Molecular cluster with the hydrogen-bond distance, in Å, listed. Acceptor/PGH_3 energy interaction values and their components (in kcal/mol) according to the ETS energy decomposition scheme. Bottom in each panel: Isosurfaces (±0.0005 au) of three largest NOCV contributions to charge deformation (differential) density $\Delta\varrho$ along with their corresponding charge (q in e) and orbital energy (ΔE in kcal/mol) assessment. Red / blue indicates inflow (gain) / outflow (loss) of electron density. Based on BLYP+D4//TZP calculations.

5. Details of spectroscopic characterization

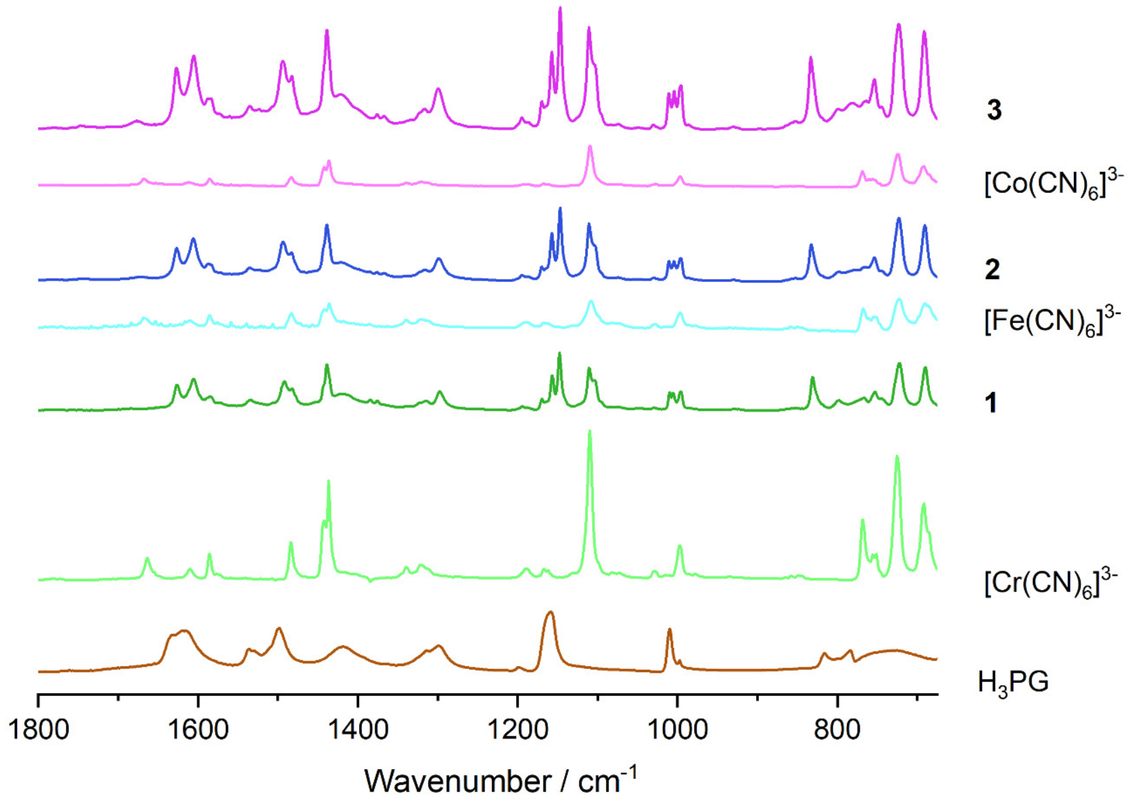

Figure S18. (a) Infrared absorption spectra of **1**-**3** in the absorption mode compared with the spectra H_3PG and $(PPh_4)_3[M(CN)_6]\cdot nH_2O$ precursor complexes showing the fingerprint range.

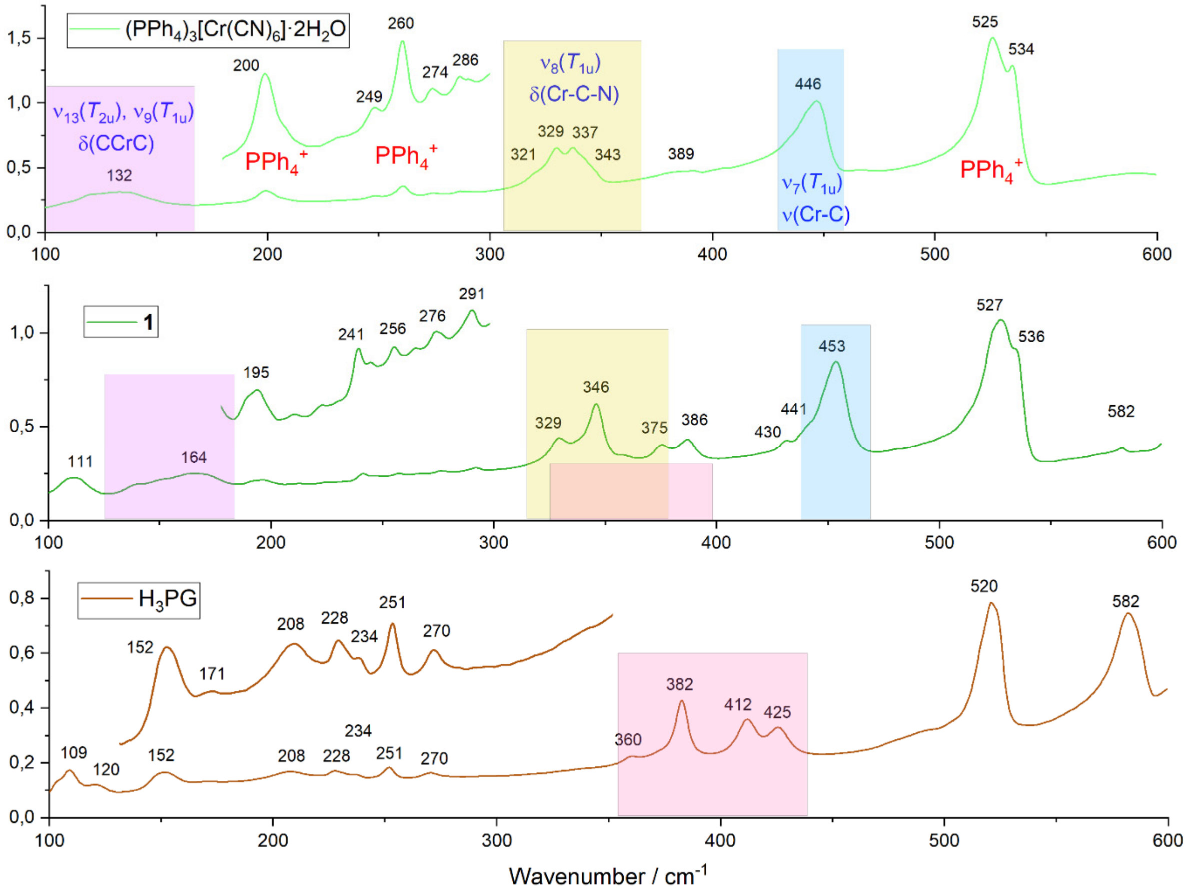

Figure S19. (a) Infrared absorption spectrum of **1** in the absorption mode compared with the spectra of H_3PG and $(PPh_4)_3[Cr(CN)_6]\cdot 2H_2O$ in the range 100-600 cm^{-1} of skeletal vibration modes. The positions of the vibration modes δ(C-Cr-C), δ(Cr-C-N) and δ(Cr-C) of $\{Cr(CN)_6\}^{3-}$ vibrations are indicated according to the Refs. [20,21]. The recognized changes in the bands occurrence and energies were assigned to the modification of components in vibrational structures due to their engagement in hydrogen-bonding networks. The positions of the bands of PPh^{4+} cations were indicated based on the computational data in Ref. [22] The presented juxtaposition is also representative for the whole series **1**-**3**.

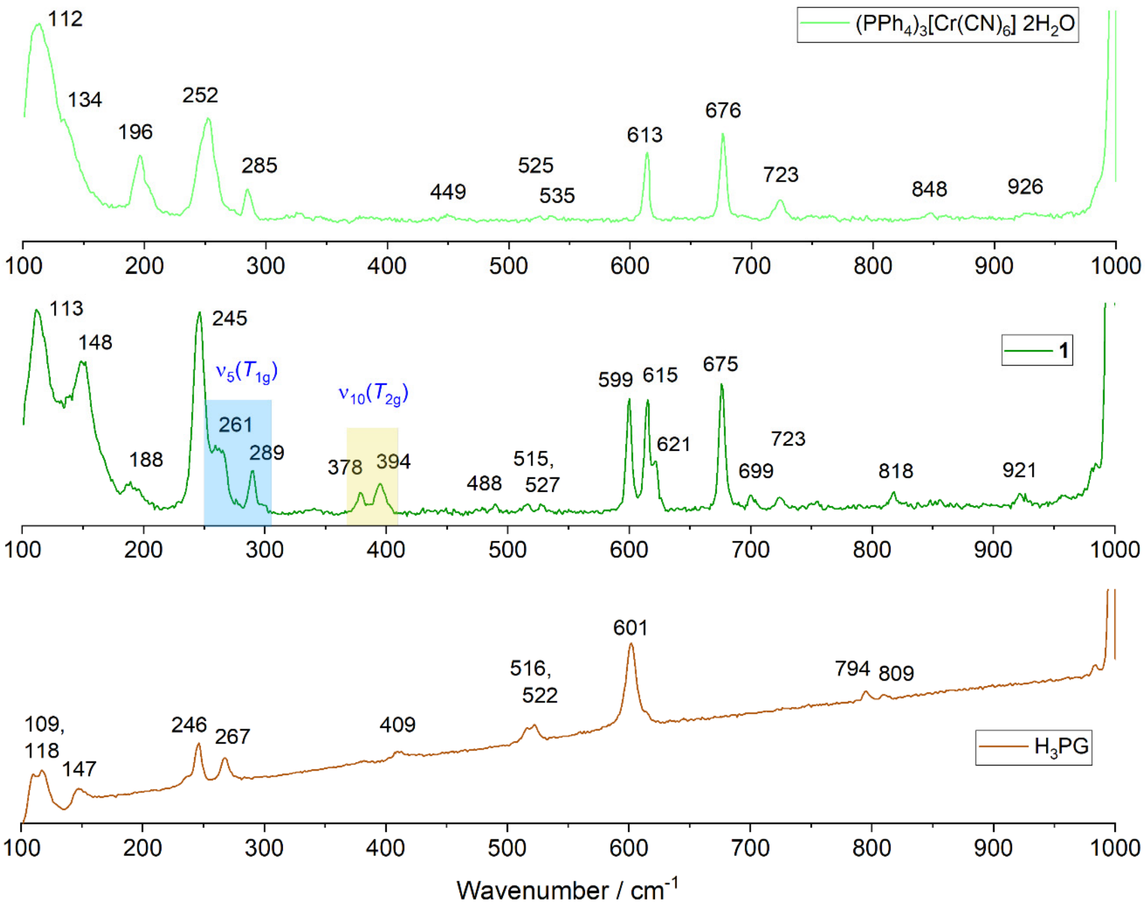

Figure S20. Raman spectrum of **1** compared with the spectra of H_3PG and $(PPh_4)_3[Cr(CN)_6]\cdot 2H_2O$ in the range 100-1000 cm^{-1}. The positions of the vibration modes $\nu_5(T_{1g})$ and $\nu_{10}(T_{2g})$ of $[Cr(CN)_6]^{3-}$ were indicated according to the Refs. [20]. The latter mode appears most probably due to the symmetry reason. The complete exact bands assignment to the molecular building blocks in **1** is not straightforward based solely on the comparison of the components spectra. The recognized changes in the bands occurrence and energies were assigned to the modification of components in vibrational structures due to their engagement in hydrogen-bonding networks.

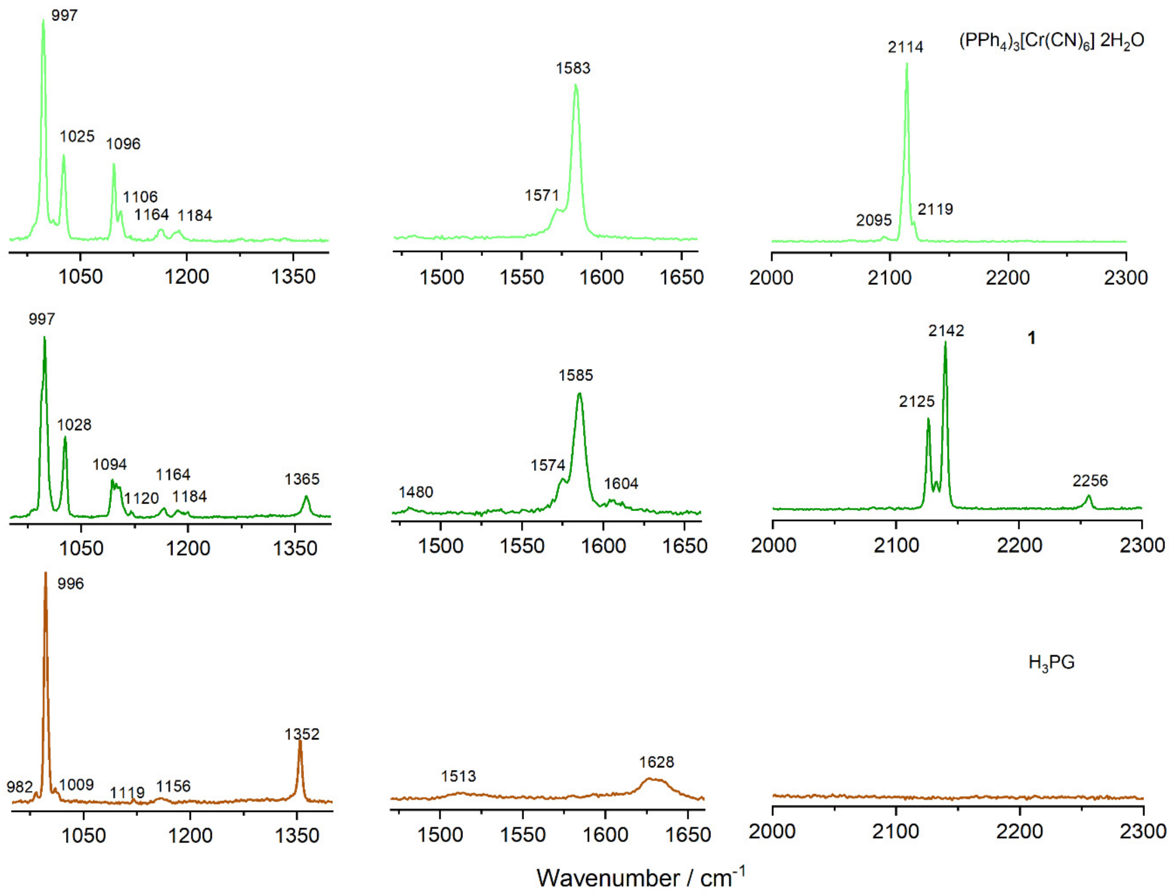

Figure S21. Raman spectrum of **1** compared with the spectra of H_3PG and $(PPh_4)_3[Cr(CN)_6]\cdot 2H_2O$ in the ranges 950-1400, 1470-1660, and 2000-2300 cm^{-1}. The bands assignment for the molecular building blocks in **1** might be tentatively done based on simple comparison. The recognized changes in the bands occurrence and energies were assigned to the modification of the components in vibrational structures, due to their engagement in hydrogen-bonding networks. Noteworthy, the ν(C≡N) stretching frequencies in the range 2000-2300 cm^{-1} confirms significant stiffening of the cyanido-ligands. See also, the modifications in the energy of the selected vibrations of H_3PG, e.g. 1365, 1480, and 1604 cm^{-1} (**1**) *vs.* 1352, 1513, and 1628 cm^{-1} (H_3PG), respectively.

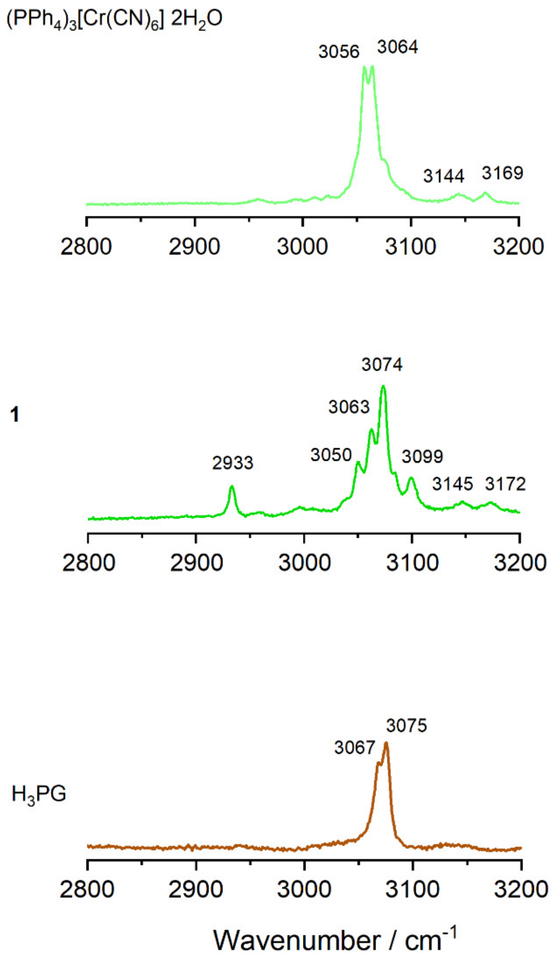

Figure S22. Raman spectrum of **1** compared with the spectra of H_3PG and $(PPh_4)_3[Cr(CN)_6]\cdot 2H_2O$ in the range 2800-3200 cm^{-1}. The bands assignment for the molecular building blocks in **1** might be tentatively done based on simple comparison.

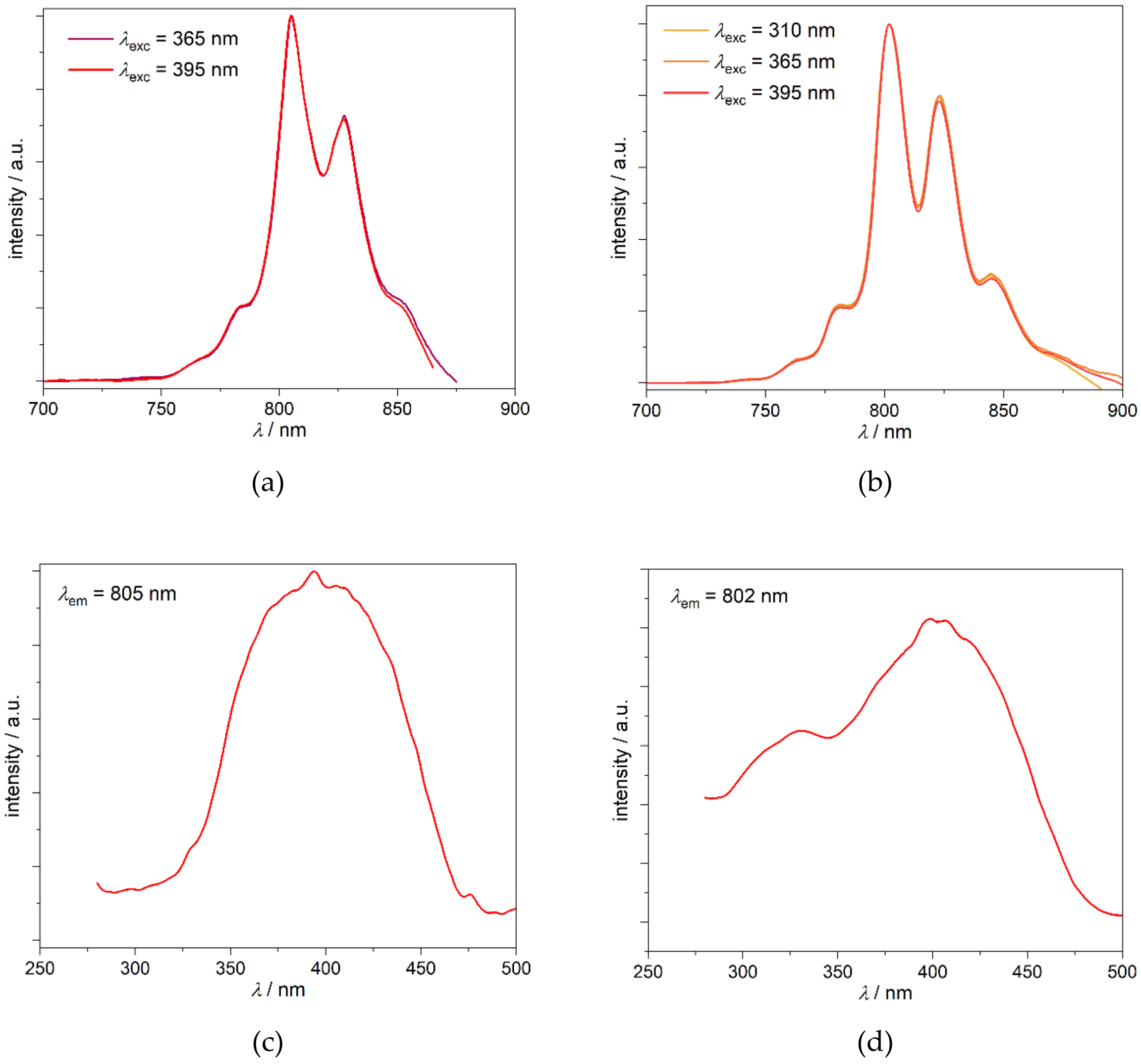

Figure S23. $^2E_g \rightarrow {}^4A_{2g}$ emission spectra of **1** (a) and $(PPh_4)_3[Cr(CN)_6]\cdot 2H_2O$ reference (b) at various excitation wavelengths in 298 K. The bottom panel show the excitation spectra of **1** (c) and $(PPh_4)_3[Cr(CN)_6]\cdot 2H_2O$ reference (d) at λ_{em} related to the highest emission intensity in emission spectra.

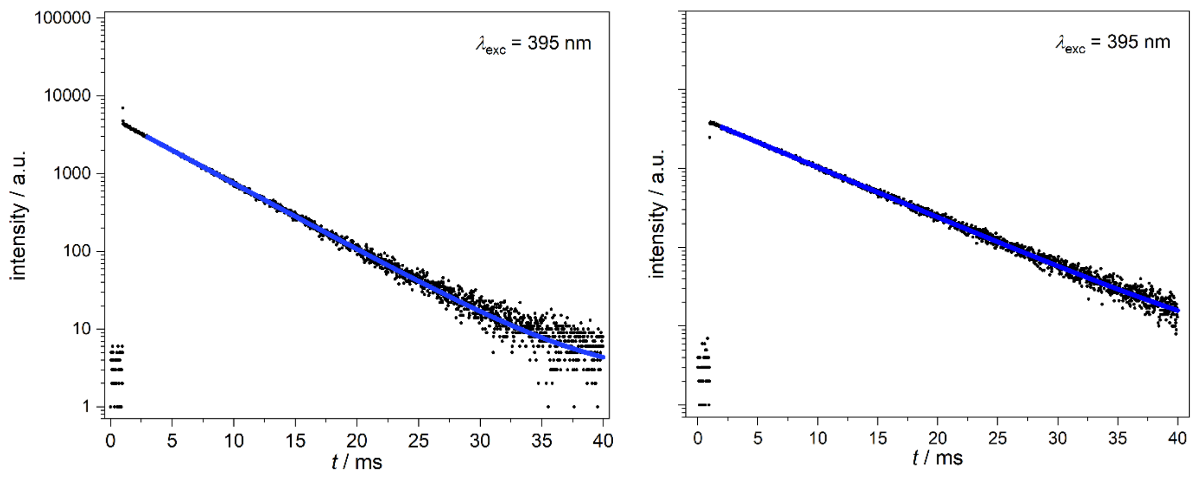

(a) (b)

Figure S24. The representative time dependent decay of the $^2E_g \rightarrow {}^4A_{2g}$ emission upon λ_{exc} = 395 nm for **1** and $(PPh_4)_3[Cr(CN)_6]\cdot 2H_2O$ reference (precursor) followed with the 810 nm (**1**) or 805 nm (ref.) emission lines together with the fits performed using the single process equation $\log R(t) = \log B_1 - t/\tau_1$. The complete of the obtained parameters is shown below including also the RT data.

LN_2

1 810 nm, τ_1 = 5.086±0.007 ms, B_1 = 2 974±5, χ^2 = 1.06308

833 nm, τ_1 = 5.088±0.007 ms, B_1 = 2 845±5, χ^2 = 1.00011

Precursor 805 nm, τ_1 = 6.809±0.010 ms, B_1 = 3 360±5, χ^2 = 1.00925

829 nm, τ_1 = 6.851±0.012 ms, B_1 = 2 135±4, χ^2 = 1.05121

853 nm, τ_1 = 6.839±0.012 ms, B_1 = 2 412±4, χ^2 = 1.03334

RT

1 805 nm, τ_1 = 4.436±0.011 ms, B_1 = 1 679±3, χ^2 = 1.01608

827 nm, τ_1 = 4.465±0.013 ms, B_1 = 1 162±3, χ^2 = 1,06577

850 nm, τ_1 = 4.479±0.014 ms, B_1 = 1 073±3, χ^2 = 1.02036

Precursor 802 nm, τ_1 = 5.549±0.016 ms, B_1 = 1 955±3, χ^2 = 0.98684

823 nm, τ_1 = 5.557±0.019 ms, B_1 = 1 301±3, χ^2 = 1,03459

845 nm, τ_1 = 5.536±0.016 ms, B_1 = 1 785±3, χ^2 = 0.95572

6. Thermogravimetric analysis

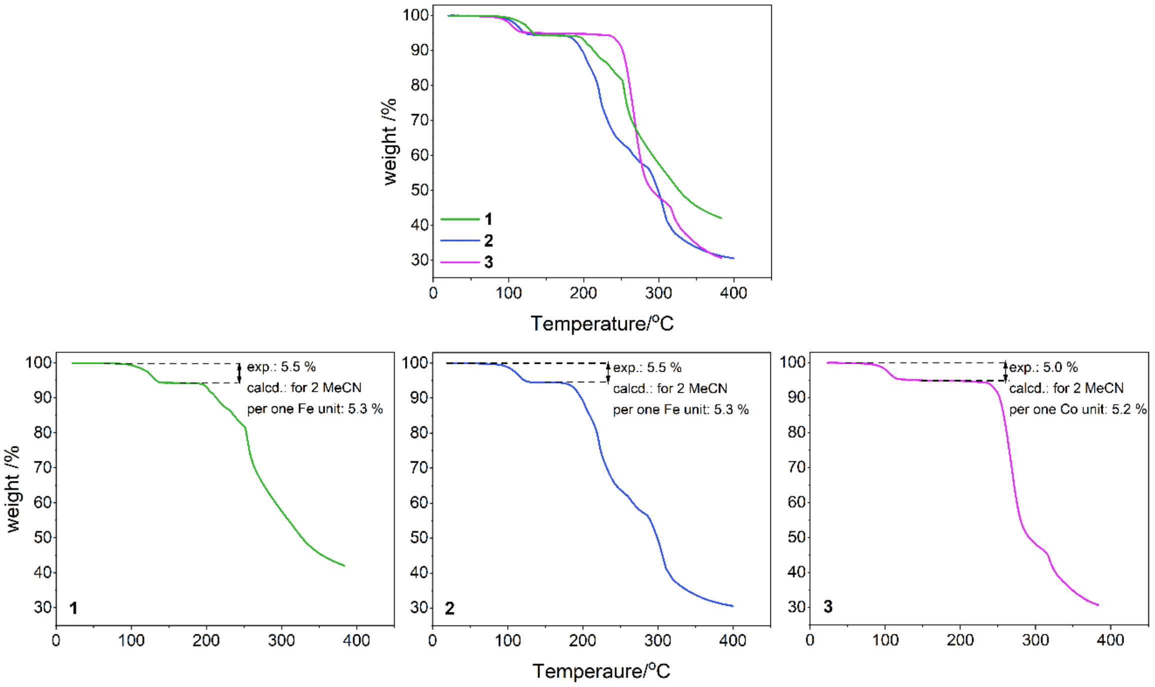

Figure S25. Thermogravimetric curves of **1–3** collected under nitrogen atmosphere with the heating rate of 1 K per minute. The upper graph contains the comparison of TG curves for all compounds while the other graphs show separately TG curves for each compound (**1**,**2** and **3**). Upon heating under nitrogen atmosphere the powder samples show stable composition up to T = 80°C (**1**) and 70°C (**2**,**3**), then exhibit small decrease of the mass up to T = 140°C (**1**), 130°C (**2**), and 120°C (**3**). These steps are assigned to the loss of two acetonitrile solvent molecules per formula unit, in agreement with the amount of solvent molecules determined by CHN analyses (described in the synthetic procedures in main text). Then, the desolvated samples show stable composition up to 190°C (**1**), 170°C (**2**), and 230°C (**3**) then undergo massive decomposition.

References

1. Dance, I.; Scudder, M.; Supramolecular Motifs: Concerted Multiple Phenyl Embraces between Ph_4P^+ Cations Are Attractive and Ubiquitous, *Chem. – Eur. J.* **1996**, *2*, 481–486.
2. Alborés, P.; Slep, L.D.; Weyhermüller, T.; Rentschler, E.; Baraldo, L.M. Exchange coupling across the cyanide bridge: structural and DFT interpretation of the magnetic properties of a binuclear chromium(III) complex. *Dalton Trans.* **2006**, 948-954.
3. Graham, M.J.; Zadrozny, J.M.; Shiddiq, M.; Anderson, J.S.; Fataftah, M.S; Hill, S.; Freedman, D.E.; Influence of Electronic Spin and Spin–Orbit Coupling on Decoherence in Mononuclear Transition Metal Complexes. *J. Am. Chem. Soc.* **2014**, *136*, 7623–7626.
4. Dubey, R.; Desiraju, G. R. Combinatorial Crystal Synthesis: Structural Landscape of Phloroglucinol:1,2-Bis(4-Pyridyl)Ethylene and Phloroglucinol:Phenazine. *Angew. Chem., Int. Ed.* **2014**, *53*, 13178–13182.
5. Coupar, P. I.; Ferguson, G.; Glidewell, C. Hydrogen-Bonded Chains in 4,4′-Dihydroxybenzophenone–4,4′-Bipyridyl (1/1) and Chains of Rings in 1,3,5-Trihydroxybenzene–4,4′-Bipyridyl (2/3). *Acta Cryst C* **1996**, *52*, 2524–2528.
6. te Velde, G.; Bickelhaupt, F. M.; Baerends, E. J.; Fonseca Guerra, C.; van Gisbergen, S. J. A.; Snijders, J. G.; Ziegler, T. Chemistry with ADF. *J. Comput. Chem.* **2001**, *22*, 931–967.
7. ADF 2019.304, SCM, Theoretical Chemistry, Vrije Universiteit, Amsterdam, The Netherlands, http://www.scm.com. (accessed 2022 -04 -21).
8. Caldeweyher, E.; Ehlert, S.; Hansen, A.; Neugebauer, H.; Spicher, S.; Bannwarth, C.; Grimme, S. A Generally Applicable Atomic-Charge Dependent London Dispersion Correction. *J. Chem. Phys.* **2019**, *150*, 154122.
9. Becke, A. D. Density-Functional Exchange-Energy Approximation with Correct Asymptotic Behavior. *Phys. Rev. A* **1988**, *38* (6), 3098–3100..
10. Lee, C.; Yang, W.; Parr, R. G. Development of the Colle-Salvetti Correlation-Energy Formula into a Functional of the Electron Density. *Phys. Rev. B* **1988**, *37*, 785–789.
11. Becke, A. D. Density-Functional Thermochemistry. III. The Role of Exact Exchange. *J. Chem. Phys.* **1993**, *98*, 5648–5652.
12. Stephens, P. J.; Devlin, F. J.; Chabalowski, C. F.; Frisch, M. J. Ab Initio Calculation of Vibrational Absorption and Circular Dichroism Spectra Using Density Functional Force Fields. *J. Phys. Chem.* **1994**, *98*, 11623–11627.
13. Santra, G.; Sylvetsky, N.; Martin, J. M. L. Minimally Empirical Double-Hybrid Functionals Trained against the GMTKN55 Database: RevDSD-PBEP86-D4, RevDOD-PBE-D4, and DOD-SCAN-D4. *J. Phys. Chem. A* **2019**, *123*, 5129–5143.
14. Klamt, A.; Schüürmann, G. COSMO: A New Approach to Dielectric Screening in Solvents with Explicit Expressions for the Screening Energy and Its Gradient. *J. Chem. Soc., Perkin Trans.* **1993**, *2*, 799–805.
15. Klamt, A. Conductor-like Screening Model for Real Solvents: A New Approach to the Quantitative Calculation of Solvation Phenomena. *J. Phys. Chem.* **1995**, *99*, 2224–2235.
16. van Lenthe, E.; Baerends, E. J.; Snijders, J. G. Relativistic Regular Two-Component Hamiltonians. *J. Chem. Phys.* **1993**, *99*, 4597–4610.
17. van Lenthe, E.; Baerends, E. J.; Snijders, J. G. Relativistic Total Energy Using Regular Approximations. *J. Chem. Phys.* **1994**, *101*, 9783–9792.
18. Mitoraj, M.; Michalak, A. Natural Orbitals for Chemical Valence as Descriptors of Chemical Bonding in Transition Metal Complexes. *J Mol Model* **2007**, *13*, 347–355.
19. Mitoraj, M. P.; Michalak, A.; Ziegler, T. A Combined Charge and Energy Decomposition Scheme for Bond Analysis. *J. Chem. Theory Comput.* **2009**, *5*, 962–975.
20. Hanuza, J.; Strek, W.; Hermanowicz, K.; Jezowska-Trzebiatowska, B.; Trabjerg, I. Spectroscopic behaviour of $Cr(CN)_6^{3-}$ ion isolated in KCl host. *J. Mol. Struct.* **1986**, *144*, 141–153.
21. Mączka, M.; Nowok, A.; Zaręba, J.K.; Stefańska, D.; Gągor, A.; Trzebiatowska, M.; Sieradzki, A. Near-Infrared Phosphorescent Hybrid Organic–Inorganic Perovskite with High-Contrast Dielectric and Third-Order Nonlinear Optical Switching Functionalities. *ACS Appl. Mater. Interfaces* **2022**, *14*, 1460–1471.
22. Kuzniak, E.; Pinkowicz, D.; Hooper, J.; Srebro-Hooper, M.; Hetmańczyk, Ł.; Podgajny, R. Molecular Deformation, Charge Flow, and Spongelike Behavior in Anion–π $\{[M(CN)_4]^{2-};[HAT(CN)_6]\}_\infty$ (M=Ni, Pd, Pt) Supramolecular Stacks. *Chem. Eur. J.* **2018**, *24*, 16302–16314.
